# Comprehensive Analysis of Disease-Related Genes in Chronic Lymphocytic Leukemia by Multiplex PCR-Based Next Generation Sequencing

**DOI:** 10.1371/journal.pone.0129544

**Published:** 2015-06-08

**Authors:** Claudia Vollbrecht, Fabian Dominik Mairinger, Ulrike Koitzsch, Martin Peifer, Katharina Koenig, Lukas Carl Heukamp, Giuliano Crispatzu, Laura Wilden, Karl-Anton Kreuzer, Michael Hallek, Margarete Odenthal, Carmen Diana Herling, Reinhard Buettner

**Affiliations:** 1 Institute of Pathology, University Hospital Cologne, Cologne, Germany; 2 Center for Integrated Oncology (CIO) Cologne-Bonn, University Hospital Cologne, Cologne, Germany; 3 Center of Molecular Medicine Cologne, University of Cologne, Cologne, Germany; 4 Institute of Pathology, University Hospital Essen, University of Duisburg-Essen, Essen, Germany; 5 Department of Translational Genomics, Cologne Center of Genomics, University of Cologne, Cologne, Germany; 6 Department I of Internal Medicine, University Hospital Cologne, Cologne, Germany; 7 Excellence Cluster for Cellular Stress Response and Aging-Associated Diseases (CECAD), University of Cologne, Cologne, Germany; Yale University, UNITED STATES

## Abstract

**Background:**

High resolution molecular studies have demonstrated that the clonal acquisition of gene mutations is an important mechanism that may promote rapid disease progression and drug resistance in chronic lymphocytic leukemia (CLL). Therefore, the early and sensitive detection of such mutations is an important prerequisite for future predictive CLL diagnostics in the clinical setting.

**Material & Methods:**

Here, we describe a novel, target-specific next generation sequencing (NGS) approach, which combines multiplex PCR-based target enrichment and library generation with ultra-deep high-throughput parallel sequencing using a MiSeq platform. We designed a CLL specific target panel, covering hotspots or complete coding regions of 15 genes known to be recurrently mutated and/or related to B-cell receptor signaling.

**Results:**

High-throughput sequencing was performed using as little as 40 ng of peripheral blood B-cell DNA from 136 CLL patients and a dilution series of two *ATM*- or *TP53*-mutated cell lines, the latter of which demonstrated a limit of mutation detection below 5%. Using a stringent functional assessment algorithm, 102 mutations in 8 genes were identified in CLL patients, including hotspot regions of *TP53*, *SF3B1*, *NOTCH1*, *ATM*, *XPO1*, *MYD88*, *DDX3X* and the B-cell receptor signaling regulator *PTPN6*. The presence of mutations was significantly associated with an advanced disease status und molecular markers of an inferior prognosis, such as an unmutated *IGHV* mutation status or positivity for ZAP70 by flow cytometry.

**Conclusion:**

In summary, targeted sequencing using an amplicon based library technology allows a resource-efficient and sensitive mutation analysis for diagnostic or exploratory purposes and facilitates molecular subtyping of patient sets with adverse prognosis.

## Introduction

Chronic lymphocytic leukemia (CLL) is an incurable and common type of adult leukemia with significant variability in clinical prognosis that is hard to predict [1, 2]. The current biological understanding is that variable courses of the disease are predominantly caused by molecular inter- and intrapatient heterogeneity of leukemic cells and the possibility of clonal disease evolution over time [2–4].

Recent whole-exome and genome sequencing studies have deciphered the mutational landscape in CLL and discovered a variety of somatic mutations and small indels in *NOTCH1*, *SF3B1*, and other candidate genes, which encode for putative and previously unknown drivers of CLL tumorigenesis [5–10]. Some of these mutations seem to be associated with prognosis, however, except for mutations and other genomic aberrations in the *TP53* gene, the clinical consequences to be taken in case a patient presents with one of these mutations, are not clarified [6, 7, 10–14].

Future risk assessment in CLL is now confronted with the need of prospective clinical trials, which systematically integrate mutation and traditional biomarker assessment to determine the parameters with a retained prognostic or predictive value, relevant to clinical practice. This has become of particular importance as new drugs, e.g. inhibitor to protein kinases PI3K and BTK, are entering clinical practice and conveying new mechanisms of treatment resistance compared to standard chemoimmunotherapy [15].

The aim of our study presented here was to develop a targeted genomic sequencing assay, being able to meet such diagnostic and clinical research needs in CLL. Targeted sequencing versus whole-genome or exome-wide massive parallel sequencing (i.e. next generation sequencing, NGS) offers the opportunity to assess genomic changes in areas of specific interest at a coverage as high as deemed appropriate for diagnostic reporting.

In comparison to traditional Sanger sequencing currently used for routine assessment of the *TP53* or *IGHV* genes, NGS allows multiplexing of samples and gene targets in one experimental setup. In addition, the possibility of automation for high-throughput sample processing further minimizes clinical laboratory efforts and final costs per gene and sample [16]. So far, only few studies have implemented targeted NGS technologies for mutation screening in CLL [17–20].

We here describe a multiplex PCR-based sequencing panel suitable for a high-throughput benchtop sequencer as represented by the Illumina MiSeq platform. In addition to genes confirmed to be mutated in previous CLL sequencing studies, such as *ATM*, *CD79B*, *DDX3X*, *FBXW7*, *MYD88*, *NOTCH1*, *SF3B1*, *TP53*, *XPO* [6, 7, 10, 21–23], we chose target genes directly or indirectly involved in the B-cell receptor (BCR) signaling pathway (*BTK*, *MAPK1*, *PIK3CA*, *PIK3CD*, *PTEN*, *PTPN6*). Using a modified chemistry setup for target enrichment and library preparation in a test cohort of 136 CLL patients and two mutated cell lines, we were able to obtain a high sequencing coverage and a low limit of mutation detection. Previously known and new mutations were detected in coding or hotspot regions of the genes *ATM*, *DDX3X*, *MYD88*, *NOTCH1*, *SF3B1*, *TP53*, *XPO1* and *PTPN6* (SHP-1), and associations between mutations and adverse prognostic markers were investigated.

Overall, our targeted NGS approach resembles a sensitive and resource efficient method for simultaneous mutation analysis of multiple gene regions on a high-throughput sequencing platform and is highly suitable to future diagnostic and clinical research purposes in CLL.

## Materials and Methods

### Clinical Samples

The study was approved by the ethical commission of the medical faculty of the University of Cologne (reference no. 13–091) and an informed written consent was obtained from all patients. Between 2012 and 2013, 136 blood samples from CLL patients were collected at the University of Cologne, Germany. All cases demonstrated typical features of CLL as defined by the International Workshop on CLL [24]. Clinical and routine laboratory parameters were retrieved from medical records. CLL-related chromosomal abnormalities were assessed by interphase fluorescence-in-situ hybridization (FISH) using commercially available probes, detecting trisomy 12 and deletions on chromosomes 6q21 (*SEC63*), 11q22.3 (*ATM*), 13q34 (*D13S319*) and 17p13.1 (*TP53*) (Abbott, Abbott Park, IL, USA). In addition, CLL immunophenotypes including CD38 and ZAP70 surface expression and the somatic mutation status of *IGHV* genes was determined as described previously [25].

B-cells were enriched by negative selection using RosetteSep-based cell removal (Stemcell Technologies, Vancouver, BC, Canada) followed by Pancoll human density centrifugation (Pan Biotech, Aidenbach, Germany).

Genomic DNA was extracted from B-cell fractions by standard column based purification (DNeasy, Qiagen, Hilden, Germany). DNA quality and quantity was assessed by gel electrophoresis.

### Library Construction and Deep Sequencing

In order to selectively amplify either hotspot or complete coding regions of the following genes *ATM*, *BTK*, *CD79B*, *DDX3X*, *FBXW7*, *MAPK1*, *MYD88*, *NOTCH1*, *PIK3CA*, *PIK3CD*, *PTEN*, *PTPN6*, *SF3B1*, *TP53* and *XPO1*, two panels containing 338 primer pairs in four separate pools were designed using the Ion AmpliSeq algorithm of Life Technologies (Table 1 and S1 Table). Amplifiable DNA was quantified by qPCR (S2 Table). Subsequently, target enrichment and library preparation followed the instructions of the “Ion AmpliSeq Library Kit 2.0” (Life Technologies) and the “NEXTflex DNA Sequencing Kit, Manual V11.12” (Bioo Scientific, Austin, TX, USA). Detailed methods are available on request (http://www.lungcancergroup.de). Briefly, a total of 40 ng genomic B-cell DNA was amplified in four separate multiplex PCR reactions per sample. All purification and size selection steps were performed with magnetic beads (Agencourt AMPure XP, Beckman Coulter, Brea, CA, USA) and a Biomek FX^p^ workstation (Beckman Coulter). Samples were diluted 10-fold before adenylation and adapter ligation. Finally, library quality was analyzed by microfluidic electrophoresis using the 2100 Bioanalyzer (Agilent Technologies, Santa Clara, CA, USA) and amplicons were quantified by qPCR (S3 Table). For sequencing, samples were pooled in an equimolar ratio. 15 pM library pools including 1% PhiX control library were prepared for sequencing according to the MiSeq System User Guide (Illumina, San Diego, CA, US). Subsequently, sequencing was carried out on a MiSeq instrument (Illumina) using the v2 chemistry as recommended by the manufacturer.

**Table 1:** Overview of the genes covered by the CLL panels.

Gene	Biological Process	Exons	Transcript ID	n Amplicons
*ATM*	DNA damage/ cell cycle control	Complete (62)	NM_000051	117
*BTK*	B-cell receptor signaling pathway	14–16	NM_000061	5
*CD79B*	B-cell receptor signaling pathway	4–5	NM_021602	2
*DDX3X*	RNA splicing and processing	7–9, 11, 14	NM_001356	6
*FBXW7*	Protein ubiquitination	6–9	NM_033632	7
*MAPK1*	MAP kinase signaling pathway	7	NM_002745	1
*MYD88*	Toll-like receptor signaling pathway	Complete (5)	NM_002468	9
*NOTCH1*	Notch signaling pathway	Complete (34)	NM_017617	71
*PIK3CA*	B-cell receptor signaling pathway	9–11, 20–21	NM_006218	10
*PIK3CD*	B-cell receptor signaling pathway	21–24	NM_005026	7
*PTEN*	AKT-mTOR signaling pathway	5–6, 9	NM_000314	7
*PTPN6* (SHP-1)	B-cell receptor signaling pathway	11–12	NM_080548	2
*SF3B1*	RNA splicing and processing	Complete (25)	NM_012433	52
*TP53*	DNA damage/ cell cycle control	Complete (9)	NM_000546	16
*XPO1*	RNA splicing and processing	12–13, 15	NM_003400	7
**Total number of amplicons**				**338**

### Estimation of Lowest Detection Rate Using Cell Line DNA Dilutions

The mantle cell lymphoma cell line, Mino (kindly provided by M. Herling, Cologne, Germany), carrying a known homozygous *TP53* mutation (c.440T>G; p.V147G; NM_000546) [26], and the AT45RM B-cell line (kindly provided by L. Chessa, Rome, Italy) containing an heterozygous *ATM* mutation (c.7792C>T; p.R2598*; NM_000051) [27] were used to evaluate the limit of detection (LoD) of our NGS approach. Cells were cultured according to standard protocols. DNA was extracted and sequenced as described above. 200 to 9,000 genomic copies of each cell line DNA were diluted in wild type DNA from human embryonic kidney cells (HEK-293, obtained from the American Type Culture Collection ATCC) harboring no known gene mutations.

### Sequencing Data Analysis

**Fig 1:**
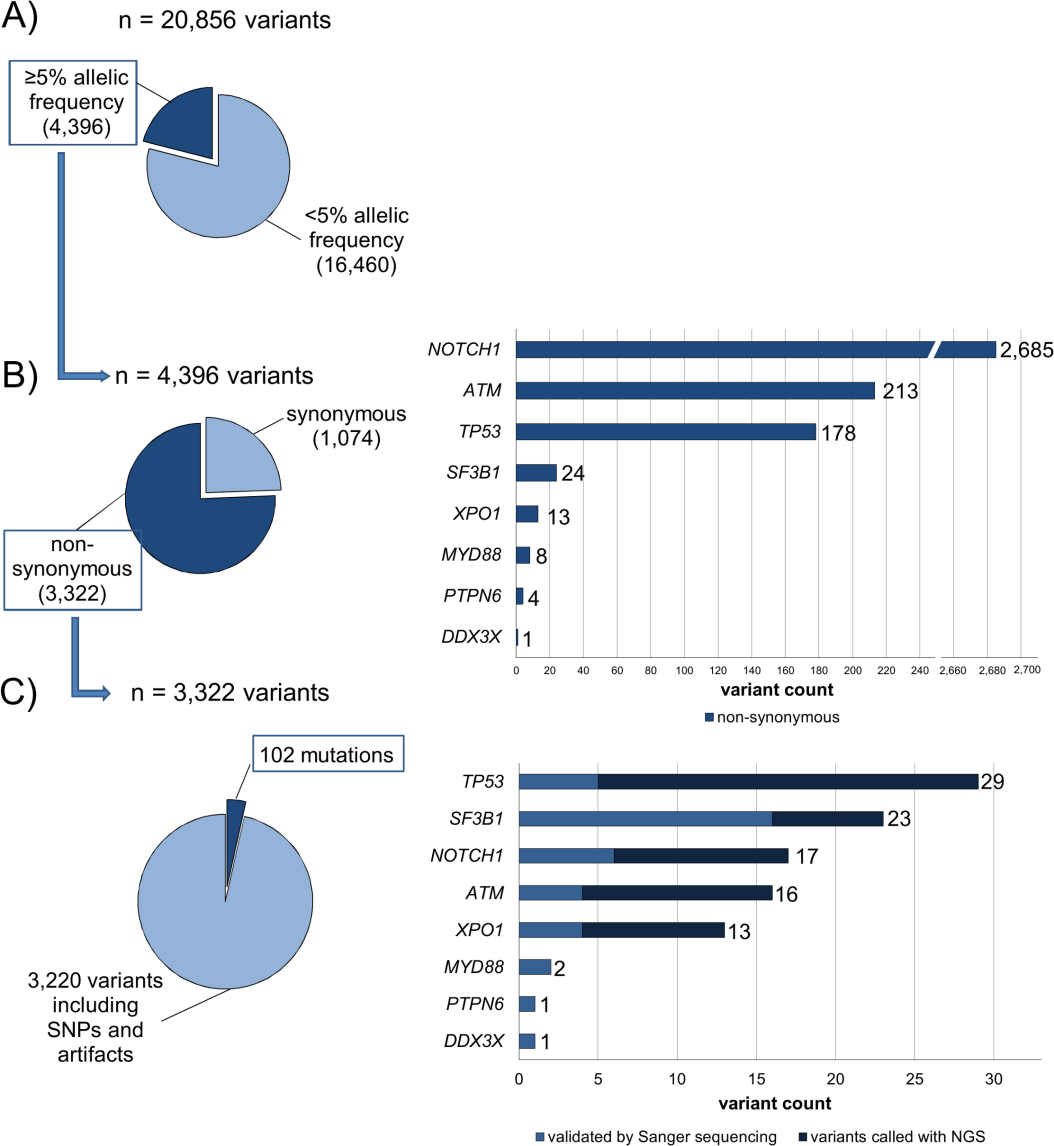
Algorithm of variant analysis. A) Variants with an allelic frequency below 5% were discarded, resulting in 4,396 variants. B) Only the 3,322 non-synonymous variants were used for further analysis. The variant count per gene is represented in the bar chart. C) Variants located in areas of high background noise and/or in homopolymeric regions, and single strand variants were visually identified in the Integrative Genomic Viewer (IGV, Broad Institute) and removed. In doubtful cases, Sanger sequencing was performed to prove or disprove an alteration. Furthermore variants without functional impact on the protein determined by at least two of four applied program algorithms as described in material and method were removed. This resulted in 102 final mutations in 60 CLL specimens.

Fastq files generated by the MiSeq Reporter Software (Illumina) were analyzed with an in-house developed bioinformatics pipeline, based on the general cancer genome analysis algorithm, which was further optimized for the diagnostic workflow [28]. Briefly, adaptor sequences were first removed from raw sequencing reads. The resulting data was then aligned against NCBI build 37 (hg19) using the Burrows-Wheeler Aligner (BWA, version 0.6.1-r104) [29] with its default settings. In order to capture longer insertion and deletions we realigned unmapped reads with the BLAST-like alignment tool (BLAT) [30, 31]. For variant calling we first determined the background error rate of the sequencer using known single nucleotide polymorphisms (SNPs): Bases diverting other than the possible two variants were counted and set into the relation to the total coverage at the location of the SNP. Finally, variants were called by testing if a mutation was not compatible with the afore mentioned error rate. For this purpose, we set the significance threshold to 0.01, which leads to a slight overcalling of the sequencing data. Spurious calls were subsequently filtered out by the following strategy: Detected variants were annotated by using the databases dbSNP (http://www.ncbi.nlm.nih.gov/SNP/) and the exome variant server (http://evs.gs.washington.edu/EVS/). Furthermore, obtained variants were analyzed for their functional impact on the protein by the MutationAssessor (http://mutationassessor.org; release 2) [32] and by implementation of the ANNOVAR algorithm [33], which combines the bioinformatic tools SIFT [34], PolyPhen2 [35] and the Mutation Taster [36]. Variants with an allelic frequency below 5%, synonymous and variants without functional impact were removed (Fig 1). Additional, visual analysis of called variants was performed by means of the Integrative Genomic Viewer (IGV, Broad Institute, Cambridge, MA, USA). Potential false positive variants, particularly in repetitive or highly homologous regions of the genome, variants in high background noise, as well as single strand variants, were either eliminated when they were clearly recognizable as artifacts, or were further re-assessed by Sanger sequencing.

### Variant Confirmation

A subset of variants, including variants with less than 100 reads, was confirmed by conventional Sanger sequencing using the BigDye Terminator v3.1 Cycle Sequencing Kit (Life Technologies) (S4 and S5 Tables). Variants that could not be confirmed were excluded from further analysis.

### Statistical Analysis

Statistical analysis for associations with clinical and/or prognostic covariates was performed for genes with mutations in multiple samples (more than 10) with predicted impact on protein function. Consequentially, the five genes *TP53*, *SF3B1*, *ATM*, *NOTCH1* and *XPO1* were tested for associations with clinical and prognostic parameters (genomic aberration, age, gender, Binet-stage, white blood count (WBC), platelets, ZAP70 and CD38 positivity and *IGHV* mutation status) as available in our dataset. Associations between mutated patient subsets and covariates were assessed applying standard statistical tests (Fisher’s exact, Pearson’s chi-square, Wilcoxon Mann-Whitney rank sum test). Correlations between linear vectors were tested via Spearman’s rho coefficients. Statistical calculations were computed in R version 3.1.0 (R Foundation for Statistical Computing, Vienna, Austria). All reported P-values were considered significant at P≤ 0.05.

## Results

### Patients’ Characteristics

We performed target-specific sequencing on purified B-cell DNA obtained from 96 men and 40 women with confirmed CLL disease, treated and followed at the University of Cologne, Germany. The majority of patients presented with previously untreated (94/70%) and/or early stage disease (Binet stage A, 73 patients/58%), at the time the sample was obtained. A subset of 41 (31%) of all patients had received a median of 2 (1–11) CLL specific treatments prior to inclusion into our study. The median time from diagnosis to sample was 41 months (0–209 months). Among patients from whom FISH analysis was available (81/60%), there was a substantial subset with deletions in chromosome 17p (13 cases/16.0%), most probably due to referral to our institution as a tertiary care center. Other prognostic markers, such as the *IGHV* mutation status, ZAP70-, CD38-surface expression, and serum thymidine kinase were distributed according to expected rates (Table 2 and S6 Table).

**Table 2:** Patient characteristics.

	Absolute
**Age at sampling (years)**	
Median (range)	63 (29–86)
**Gender**	
Male	96
Female	40
**Binet stage (n = 127/136)**	
A	73
B	30
C	24
**Treatment status (n = 135/136)**	
Untreated	94
Treated	41
**White blood count [10^9^/L]**	
Median (range)	48.8 (10.8–483.8)
***IGHV*** **somatic mutation status (n = 124/136)**	
Mutated	59
Unmutated	65
**Serum thymidine kinase (n = 78/136)**	
>10U/L	50
Median (range)	16.1 (3.5–330.0)
**ZAP70 expression (n = 86/136)**	
Positive	30
Negative	56
**CD38 expression (n = 84/136)**	
<30%	56
≥30%	28
**FISH positivity (hierarchical model, n = 81/136)**	
Del13q as sole abnormality	35
Trisomy 12	7
Del11q	9
Del17p	13
Normal	20
**Median time from diagnosis to sampling (range) in months**	61.5 (0–296.1)

### High Levels of Target Coverage and Low Limit of Mutation Detection

All samples successfully completed targeted sequencing in a total of five runs, each producing an average output of 15.37x10^6^ reads and 4.7 gigabases (S7 Table). 15 genes covered by 338 amplicons demonstrated a mean coverage per exon in a range of 0 to 7,156 reads. Only for five exons (3%; *ATM* exon 20, *NOTCH1* exon 27, *SF3B1* exon 5 and 11, *TP53* exon 11) the mean read count was less than 100, but 83% of targeted exons were covered by more than 500 reads (S1 Fig).

Two cell lines (Mino, AT45RM) with known mutations in *TP53* (exon 5) or *ATM* (exon 53) were selected as positive controls to estimate the lowest detection rate of our targeted NGS method. Analyzing fractional dilutions of mutated cell line DNA (5% to 100%), the allelic frequency of the *TP53* and *ATM* mutations detected by NGS followed a linear relationship with increasing amounts of tumor DNA (S2 Fig, P≤0.003, rho 1.000). We unambiguously identified the homozygous *TP53* mutation p.V147G in a background of 95% wild type DNA and the heterozygous *ATM* mutation p.R2598* in up to 90% wild type DNA background (obtained allelic frequency: 2% and 8%, respectively). Therefore, our NGS method obtained an adequate low LoD to uncover small subsets of mutated CLL cells due to clonal heterogeneity.

Sequencing data analysis resulted in a total of 4,396 variants after raw data alignment and first background removal (Fig 1A and 1B). Exclusion of sequencing errors, synonymous variants, variants without functional impact, and SNPs, led to 102 mutations predicted to affect protein function by at least two of four applied program algorithms as described in material and methods [32, 33]. These 102 mutations including 83 missense mutations, 12 deletions, 6 nonsense mutations, and 1 insertion were detected in eight genes and 60 out of 136 CLL samples (Fig 1C and S8 Table). In the remaining 76 patients (56%) no variants could be identified.

Fastq files are available at European Nucleotide Archive (ENA; http://www.ebi.ac.uk/ena/data/view/PRJEB9036).

### Multiplex PCR-based NGS Detects Variants in CLL-Related Genes

The highest frequency of mutations was obtained for *TP53* and *SF3B1* followed by *NOTCH1*, *ATM* and *XPO1*, whereas *MYD88*, *PTPN6* and *DDX3X* showed only two or one variant, respectively (Fig 1C). No mutations were found in *BTK*, *CD79B*, *FBXW7*, *MAPK1*, *PIK3CA*, *PIK3CD* and *PTEN*.

**Fig 2:**
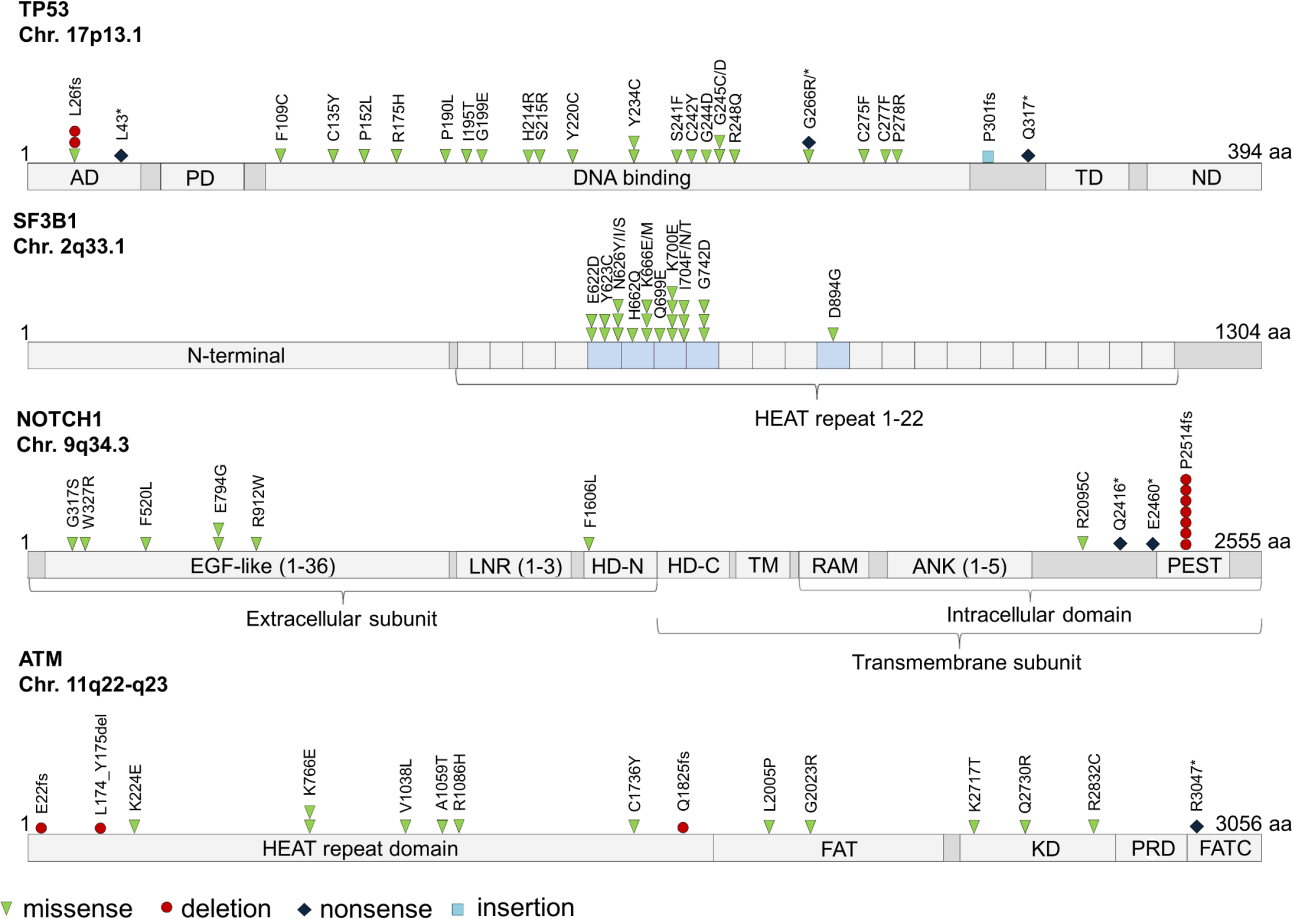
Alteration type, number of occurrence and location of detected mutations in *TP53*, *SF3B1*, *NOTCH1* and ATM are shown. *TP53*: AD activation domain (amino acid 1–50); PD proline-rich domain (amino acid 63–97); TD tetramerization domain (amino acid 323–356); ND negative regulation domain (amino acid 363–393); *SF3B1*: The majority of *SF3B1* alterations were clustered in the region encoding the highly conserved HEAT (huntingtin, elongation factor 3, protein phosphatase 2A, target of rapamycin 1) repeats 5–8. Only one alteration occurred in the N-terminal (amino acids 1–450), domain, which is an important docking or binding domain for numerous splicing factor partners like U2AF1/2, and cyclin E. *NOTCH1*: (EGF)-like epidermal growth factor repeats (amino acid 20–1426), LNR Lin-12 NOTCH repeats (amino acid 1449–1571), HD-N/C heterodimerization domain (N-terminus; C-terminus), RAM RAM domain, ANK ankyrin repeat domain (amino acid 1927–2089); PEST Pro-Glu-Ser-Thr motif for degradation (amino acid 2507–2526); *ATM*: FAT FRAP-ATM-TRRAP (amino acid 1960–2566), KD protein kinase domain (amino acid 2712–2962), PRD PIKK-regulatory domain (amino acid 2961–3025), FATC FAT-c-term domain (amino acid 3024–3056); aa amino acid

**Fig 3:**
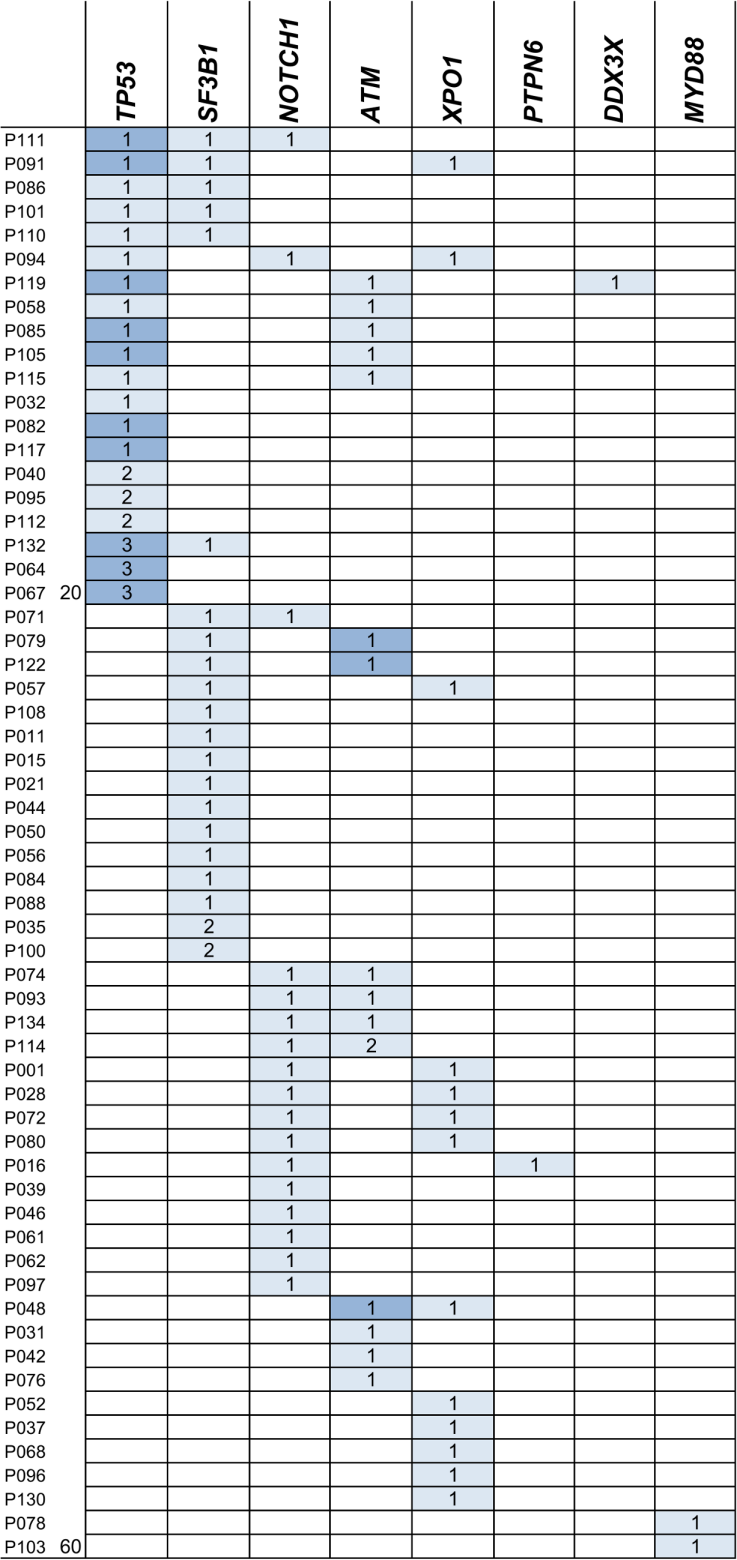
Genetic profile of 60 CLL samples carrying gene mutations determined by NGS. Each row represents the variants of one patient, each column summarizes the mutations occurring in one specific gene. Per each gene the number of mutations is given per patient. Dark blue samples indicate patients with aberration on chromosome 11 (del11q) for *ATM* mutated cases or on chromosome 17 (del17p) for *TP53* mutated cases, determined by FISH.

A total of 16 *ATM* mutations appeared in 15 of the 136 CLL patients (11%) and were evenly distributed over the entire gene (Fig 2). Interestingly, most of the samples harboring an *ATM* mutation showed at least one additional mutation in another gene (Fig 3). Three *ATM* mutated patients had also a deletion of *ATM* in the second allele, as assessed by FISH.

Nearly 98% (2,719) of detected *NOTCH1* variants turned out to be SNPs or sequencing errors. The remaining 17 mutations occurred in 17 patients (13%). Seven patients (5%) exhibited the previously reported p.P2514fs mutation, located in the PEST domain encoded by exon 34 [6]. Six variants were located in the NOTCH extracellular part (NEC, 6/17 variants, 35%). Interestingly, one patient exhibited a missense mutation located in exon 26 (p.F1606L) affecting the heterodimerization domain (HD) of the NOTCH1 protein.

*TP53* was the second most frequently mutated gene with 20 of 136 patients (15%) harboring a total of 29 mutations. Most of them (21/29; 72%) occurred in exon 6 to 8 and were identified to disrupt the TP53 DNA binding function. Ten *TP53* mutated patients had also a deletion of *TP53* in the second allele, verified by FISH.

In agreement to previous data from Wang *et al*. *SF3B1* showed a mutation frequency of 15% (21/136 patients) clustering in exon 14 to 16 [10]. The most frequent mutation was determined as p.K700E in exon 15 (4/23, 17%). Typical and functionally relevant exon 15 mutations in *XPO1* occurred in 13 patients (10%, p.E571I/K/Q) [6, 18].

Furthermore, we found two *MYD88* mutations (p.V217F in exon 3 and p.L265P in exon 5) in two of the 136 patients (2%).

Only one patient (1%) exhibited a mutation in exon 9 of *DDX3X* (p.T275P) and one patient in exon 11 of *PTPN6* (p.V451M). The latter one was located in the highly conserved catalytic protein-tyrosine phosphatase domain of the growth factor regulator SHP-1 *(PTPN6)* and occurred with an allelic frequency of 51%.

### Variants Detected by NGS Associate with Clinical and Prognostic Parameters

Genes with mutations in at least ten samples obtained by our analysis, i.e. *TP53*, *SF3B1*, *NOTCH1*, *ATM* and *XPO1*, were tested for associations with clinical and prognostic parameters, as available in our dataset. The majority of patients without any DNA alteration detected by our sequencing panel presented significantly more frequently with early stage (Binet A/B, 47/53, 89%; P = 0.03) or previously untreated CLL (42/53, 79%; P = 0.001) at the time of sampling. Presence of mutations in *NOTCH1*, *SF3B1*, *TP53* and *XPO1* was associated with at least one unfavorable prognostic marker such as an unmutated *IGHV* gene status or positivity for ZAP70 or CD38 (Table 3). *SF3B1* mutated patients were significantly more frequent of male gender (20/96 males vs. 1/40 females, P = 0.008) and *IGHV* unmutated (15/21, 72% vs. 5/21, 24%, P = 0.03) than *SF3B1* wild type cases (S3 Fig). In the untreated patient cohort, the presence of *SF3B1* mutations significantly correlated with positivity for CD38 assessed by flow cytometry (P = 0.03) (S4 Fig).

**Table 3:** Statistical correlations between gene mutation status and clinical and biological parameters.

	Mutated Gene	Clinical/Prognostic Parameter	P-Value
**Overall Cohort**	*SF3B1*	Male sex	0.00759
	*SF3B1*	Unmutated *IGHV*	0.03029
	*SF3B1*	Decreased platelet count	0.02467
	*TP53*	Binet stage	0.00724
	*TP53*	Chromosome 17p deletion	0.00000
	*TP53*	Unmutated *IGHV*	0.03842
	*TP53*	Treatment status	0.00071
	*XPO1*	Unmutated *IGHV*	0.00015
	*XPO1*	Increased White blood count (WBC)	0.00070
**Untreated Cohort**	*NOTCH1* (p.P2514fs)	Unmutated *IGHV*	0.03646
	*SF3B1*	CD38 positivity	0.03096
**Treated Cohort**	*XPO1*	ZAP70 positivity	0.02362

Similarly, an unmutated *IGHV* status occurred more frequently in *TP53* mutants (15/20, 75% vs. 4/20, 20%, P = 0.04) and in untreated patients with a *NOTCH1* PEST domain mutation (4/4, 100% vs. 0/4, 0%, P = 0.04), compared to their wild type counterparts.

Patients with mutations in *XPO1* exhibited significantly increased WBC, possibly reflecting the proliferative capacity of CLL cells, compared to patients without *XPO1* mutations (mean: 134 vs. 65 x10^9^/L, *P*<0.001). In treated patients, positivity for ZAP70 was significantly overrepresented in patients with a mutated *XPO1* gene (P = 0.02).

Only *TP53* mutations were found to be enriched in treated versus untreated patients (12/41, 29% vs. 8/95, 8%, *P*<0.001), indicating a possible selection of this genetic alteration due to prior treatments. Further, patients with *TP53* mutations exhibited significantly more frequent deletions in the second allele on chromosome 17p, resulting in a complete disruption of the TP53 protein function (*P*<0.001) (Table 3).

## Discussion

CLL is a socioeconomically relevant disease of older adults with a currently rapidly changing field of new drugs entering clinical practice and an evolving discovery of genomic mutations with major clinical relevance [3, 15]. Future diagnostics and research in CLL and cancer in general will require the implementation of mutational screening assays, which are resource efficient, sensitive, and rapidly adaptable to clinical and scientific needs.

Here, we present a targeted sequencing assay, which combines library and sequencing chemistry beyond the boundaries of manufacturers. For this approach we optimized target amplification, sequencing output and data analysis for routine application. We performed a multiplex PCR-based library amplification combining an Ion AmpliSeq primer design (Life Technologies) with a modified library preparation chemistry that allows sequencing on an Illumina instrument. Our assay targeted the complete coding regions of five most frequently mutated “CLL-genes” (*ATM*, *MYD88*, *NOTCH1*, *SF3B1* and *TP53*) [6, 7, 10] and additionally ten genes with a more exploratory driven interest, e.g. the kinase domains of the drug targets *BTK* and *PIK3CD* [15]. Our method is performable within three days from sample DNA extraction to data analysis and offers suitable flexibility by the replacement or addition of target regions during primer design.

While targeted NGS offers the advantage to assess multiplexed samples and genes in one experimental setup—thus being relatively cost-efficient compared to Sanger sequencing-, one disadvantage is that the ability to detect mutations in a distinct gene depends on the achieved coverage/depth of reads in this specific region. Coverage and sequencing depth can vary substantially, depending on the gene region itself (e.g. GC rich, homopolymers, etc.), enzyme chemistry and sequencing platform. With our technology we were able to cover 83% of targeted exons with a minimum coverage of 500 reads. Only for five exons the mean number of reads was below 100, a threshold under which we would consider calling of mutations not possible for diagnostic purposes and apply either repeated NGS or Sanger sequencing.

Most studies on targeted CLL sequencing published so far have implemented NGS methods without giving details on the performance of the technology. Sutton *et al*. are the first to report details on assay quality and analytical requirements of targeted NGS results using the HaloPlex probe technology from Agilent in CLL [20]. This technology offers the advantage of target specific probe hybridization without PCR amplification. They investigated a set of 188 patients with poor prognostic features for gene alterations in *ATM*, *BIRC3*, *KLHL6*, *MYD88*, *NOTCH1*, *POT1*, *SF3B1 TP53*, and *XPO1*. For final analysis, they only included patients, for which they obtained at least 100 reads for 80% of the targeted bases (96% of their samples). Thus, their assay achieved reasonable quality results in terms of coverage and uniformity of read depth, comparable to ours. As discussed by the authors, cutoffs currently chosen for quality parameters to evaluate targeted NGS data are more or less arbitrary. Further studies are needed to standardize and harmonize such parameters for comparability of different datasets and clinical implementation.

Another difficulty in studies implementing high-throughput NGS in a clinical setting is the necessity to distinguish tumor acquired mutations from germline or non-tumor-specific variants. Non-tumor tissue biopsies for DNA comparison are difficult to obtain in clinical routine and additional sequencing is cost intensive. Most targeted sequencing studies in CLL published so far filtered their data according to variant information available from public databases such as dbSNP (http://www.ncbi.nlm.nih.gov/SNP/) or COSMIC (http://cancer.sanger.ac.uk/cosmic). Similarly, we applied a systematic annotation of variants detected by our assay for potential SNPs listed in dbSNP and the Exome Variant server (EVS). However, not all genetic variants described in these databases are listed as SNPs of healthy individuals in the general population, for example the activating mutation p.L858R in exon 21 of the EGF receptor gene (rs121434568). It is also conceivable, that low frequency SNPs with functional relevance on a protein level might be relevant for the CLL pathophysiology. Therefore—instead of eliminating all variants found in dbSNP or EVS—we prioritized our data for variants with functional impact on a protein level as assessed by two of four implemented program tools (MutationTaster, MutationAssessor, SIFT, Polyphen) [32, 34–36]. Only SNPs reported as benign were eliminated from the analysis. Some of the mutations detected in our study occur with approximately 100% or 50% allele frequency but show additionally a deletion on the second allele. Therefore, these mutations cannot necessarily be determined as SNPs. Hence, it is not excluded that our final list of mutations still contains variants which are not CLL-specific but so far unproven germline SNPs. For example, we describe a p.V451M mutation in the catalytic phosphatase domain of SHP-1 (*PTPN6*) which was detected at an allelic frequency of 51% and predicted to impair protein function [5, 6, 18, 37, 38]. This variant has a dbSNP entry (rs62621988) but no information about the allele origin or the clinical significance. It was described at a rare frequency of 0.0005 in the 1000 genomes project (http://www.1000genomes.org). Although allelic frequency and location in a highly conserved region point to a potential SNP, the functional relevance of this alteration is still interesting to report. SHP-1 (*PTPN6*) is a known repressor of BCR signaling. Therefore inactivating mutations could constitutively activate BCR signaling in CLL cells and therefore influence disease development and outcome [39, 40].

In general, the mutation rates obtained by our NGS assay for genes known to be mutated in CLL (*TP53*, *SF3B1*, *ATM*, *NOTCH1*, *XPO1*, *MYD88*, *DDX3X*) are comparable to other studies [6–8, 10, 13, 17–19]. Most of the mutations detected are located at typical hotspot locations, such as the p.K700E and p.G742D mutations in *SF3B1* (predominantly found in male patients), the p.L256P mutation in *MYD88*, *the* p.T275P mutation in exon 9 of *DDX3X*, or the p.E571K mutation in *XPO1* [6, 7, 10, 18, 41]. One CLL case exhibited a *MYD88* p.V217F mutation, an alteration previously described in diffuse large B-cell lymphoma (DLBCL) by Ngo *et al*. [41].

Targeted NGS studies in CLL published to date frequently omitted sequencing of *ATM*, due to the lack of hotspots regions in this relatively large gene and size limitations of their assay [13, 17–19]. In our hands, *ATM* sequencing within a larger gene panel was feasible and mutations detected at a rate of 11%, comparable to the 12% rate reported by Austen and colleagues [22]. Interestingly, we found *ATM* variants occurring more frequently in combination with other variants, in particular with *NOTCH1* or *TP53* (Fig 3), an aspect also confirmed by the study of Sutton *et al*., described above. For *NOTCH1* our data analysis obtained a high incidence of non-functional variants, which might be attributable to technical issues during target enrichment and/or sequencing, e.g. by polymerase reading errors in GC- or homopolymeric regions. Beside variants in the EGF-like and PEST domains, we detected a gain-of-function mutation (p.F1606L) in the HD domain of the NOTCH1 extracellular subunit. Only the p.P2514 frameshift deletion in the PEST domain revealed a significant correlation with an unmutated *IGHV* status (Table 3), indicating that these mutations are preferentially enriched in CLL patients with adverse prognosis.

One advantage of NGS technologies for mutation analysis is that the achievement of a high sequencing coverage allows the more sensitive determination of small subclones carrying mutations. It has been demonstrated that such subclones can evolve over time and drive CLL progression and transformation [4, 42]. In our assay, the allelic frequency of mutations ranged from 5% to 100%. The smallest clonal fraction was determined for two *NOTCH1* mutations (p.W327R; 4,765 reads and p.F1606L; 166 reads) and one *TP53* mutation (p.Y234C; 2,635 reads). Furthermore, sequencing of mutated cell lines allowed us to estimate the low LoD of our method, which depicted 5% of *TP53* mutated cells diluted in wild type background at a 2% allelic frequency. Thus, our method would be clearly able to pick up small mutated subclones in CLL, presumably beyond the detection limit of Sanger sequencing. This compares to other NGS studies reporting a sensitivity or LoD of targeted NGS at 2–3% [17, 18].

In conclusion, we have developed a targeted NGS panel and high-throughput assay for mutation analysis in CLL, which is resource-efficient and highly sensitive for the detection of low frequency alleles and fast enough to be applicable to clinical decision processes. Applying a systematic functional data assessment, we found various alterations including known hotspot mutations and one interesting *PTPN6* mutation in the BCR, without the need of non-tumor DNA sequencing. Our NGS methodology can be easily translated to molecular diagnostics of other types of cancer and may pave the way for a fast-throughput combination of morphological and molecular diagnostics in hematologic and non-hematologic malignancies.

## Supporting Information

S1 Fig Median and mean read count per exon for 167 exons of the 15 genes are shown. Results reflect data from five NGS runs.
(TIF)

S2 Fig Linear relationship of mutation rate and allele frequency detected by NGS. Sequencing of two dilution series of cell line DNA with a known A) homozygous *TP53* c.440T>G; p.V147G mutation (Mino cell line) and B) heterozygous *ATM* c.7792C>T; p.R2598* mutation (AT45RM cell line) demonstrated a linear relationship of the fractional dilution rate and the mutation allele frequency obtained by NGS. Further, the data point to the detection limit achieved by our NGS approach by detecting at least 214 *ATM* mutated AT45RM cells in a background of 2,036 wild type HEK-293 cells and 214 *TP53* mutated Mino cells in a background of 4,071 wild type HEK-293 cells.
(TIF)

S3 Fig Associations of *SF3B1* and *TP53* mutations with clinical and prognostic parameters are shown. A) *SF3B1* mutated patients were mainly *IGHV* unmutated, in contrast to *SF3B1* wild type patients that showed a normal mutated *IGHV* status (P = 0.03). B) *SF3B1* mutated patients were significantly more of male gender (P = 0.008). C) *TP53* mutations were found particularly more frequent in intermediate and advanced stage with a need for treatment (Binet stage B/C) compared with patients in an early stage (Binet stage A) (P = 0.008). D and E) *TP53* mutations were also frequently more detected in treated patients (P<0.001) and in patients with genomic aberrations on chromosome 17 (del17p) (P<0.001). ND not determined.
(TIFF)

S4 Fig The presence of functional relevant mutations in *SF3B1* and *XPO1* was associated with the unfavorable prognostic marker like positivity for ZAP70 or CD38. A) *SF3B1* mutated untreated patients showed an increased CD38 expression (p<0.04). B and C) Patients harboring *XPO1* mutations showed an increased WBC (p<0.001) and treated patients presented a higher ZAP70 expression compared to their wild type counterparts (p<0.03).
(TIF)

S1 Table Target regions of the CLL panel 1 and 2. (DOCX)

S2 Table Parameters for the *HFE* qPCR. DNA quantification was done using native DNA from HEK-293 (human embryonic kidney) cells without known gene mutations; all samples were measured in duplicates.
(DOCX)

S3 Table PCR parameters for quantification of the constructed libraries by qPCR. A) PCR components; B) PCR conditions. Amplicon library quantification was performed with 5-fold dilutions of PhiX Control V3 (Illumina, San Diego, CA, USA) in a range from 0.064 up to 40 pM as reference standard. The library samples were diluted 1:4000 and measured in duplicates.
(DOCX)

S4 Table Primer used for Sanger sequencing validation. (DOCX)

S5 Table Parameters for PCR and Sanger sequencing. A1 and 2) Components and conditions for amplification of target regions by PCR; B1 and 2) Components and conditions for Sanger sequencing reaction.
(DOCX)

S6 Table Clinical information of the patients analyzed in this study. (XLS)

S7 Table Run parameters from the five MiSeq sequencing runs. (DOCX)

S8 Table Complete list of detected mutations. Bold mutations were confirmed by Sanger sequencing. fs frame shift; * stop gained; Freq frequency; Cov coverage; dbSNP single nucleotide variants database.
(DOCX)
