# Supplementary figures and images for: Comprehensive Analysis of Disease-Related Genes in Chronic Lymphocytic Leukemia by Multiplex PCR-Based Next Generation Sequencing
Source: PLoS One. 2015 Jun 8;10(6):e0129544. doi: 10.1371/journal.pone.0129544 (PMC4459702; doi:10.1371/journal.pone.0129544)

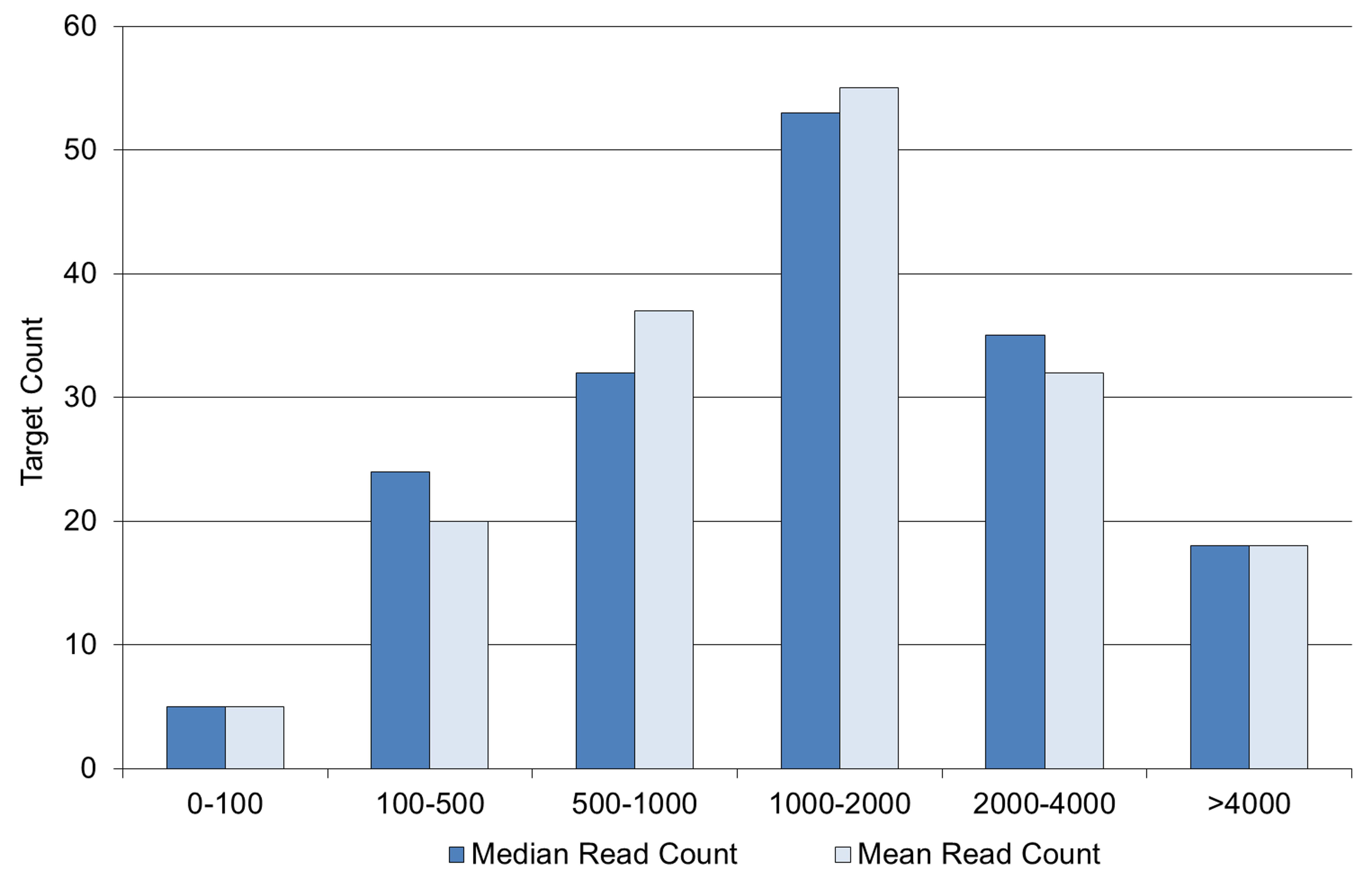

Supplement: S1 Fig — Results reflect data from five NGS runs. (TIF) [file pone.0129544.s001.tif]

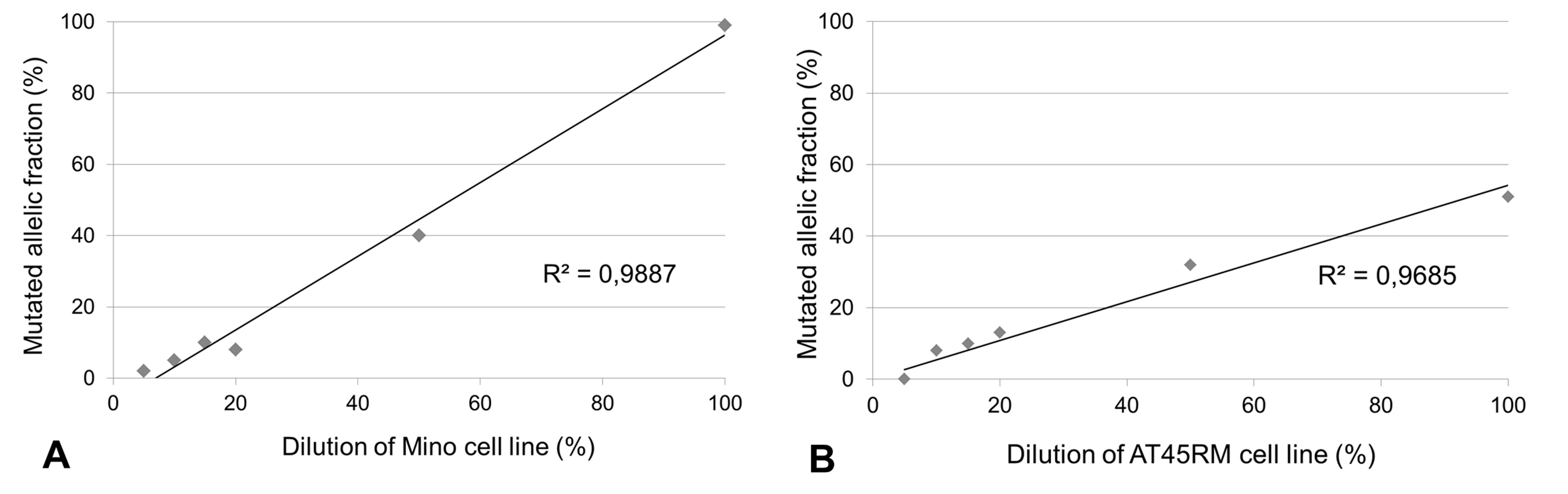

Supplement: S2 Fig — Sequencing of two dilution series of cell line DNA with a known A) homozygous TP53 c.440T>G; p.V147G mutation (Mino cell line) and B) heterozygous ATM c.7792C>T; p.R2598* mutation (AT45RM cell line) demonstrated a linear relationship of the fractional dilution rate and the mutation allele frequency obtained by NGS. Further, the data point to the detection limit achieved by our NGS approach by detecting at least 214 ATM mutated AT45RM cells in a background of 2,036 wild type HEK-293 cells and 214 TP53 mutated Mino cells in a background of 4,071 wild type HEK-293 cells. (TIF) [file pone.0129544.s002.tif]

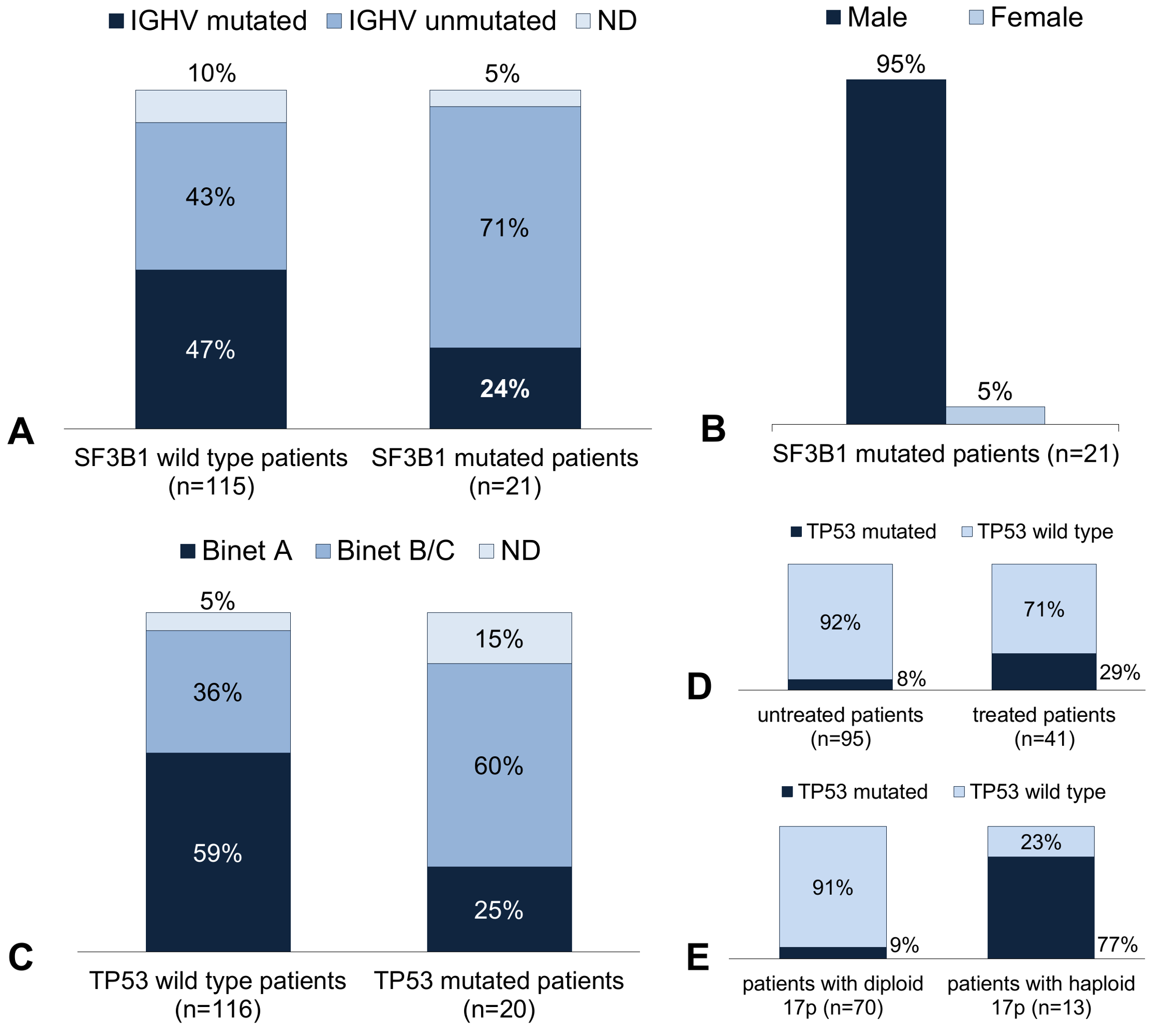

Supplement: S3 Fig — A) SF3B1 mutated patients were mainly IGHV unmutated, in contrast to SF3B1 wild type patients that showed a normal mutated IGHV status (P = 0.03). B) SF3B1 mutated patients were significantly more of male gender (P = 0.008). C) TP53 mutations were found particularly more frequent in intermediate and advanced stage with a need for treatment (Binet stage B/C) compared with patients in an early stage (Binet stage A) (P = 0.008). D and E) TP53 mutations were also frequently more detected in treated patients (P<0.001) and in patients with genomic aberrations on chromosome 17 (del17p) (P<0.001). ND not determined. (TIFF) [file pone.0129544.s003.tiff]

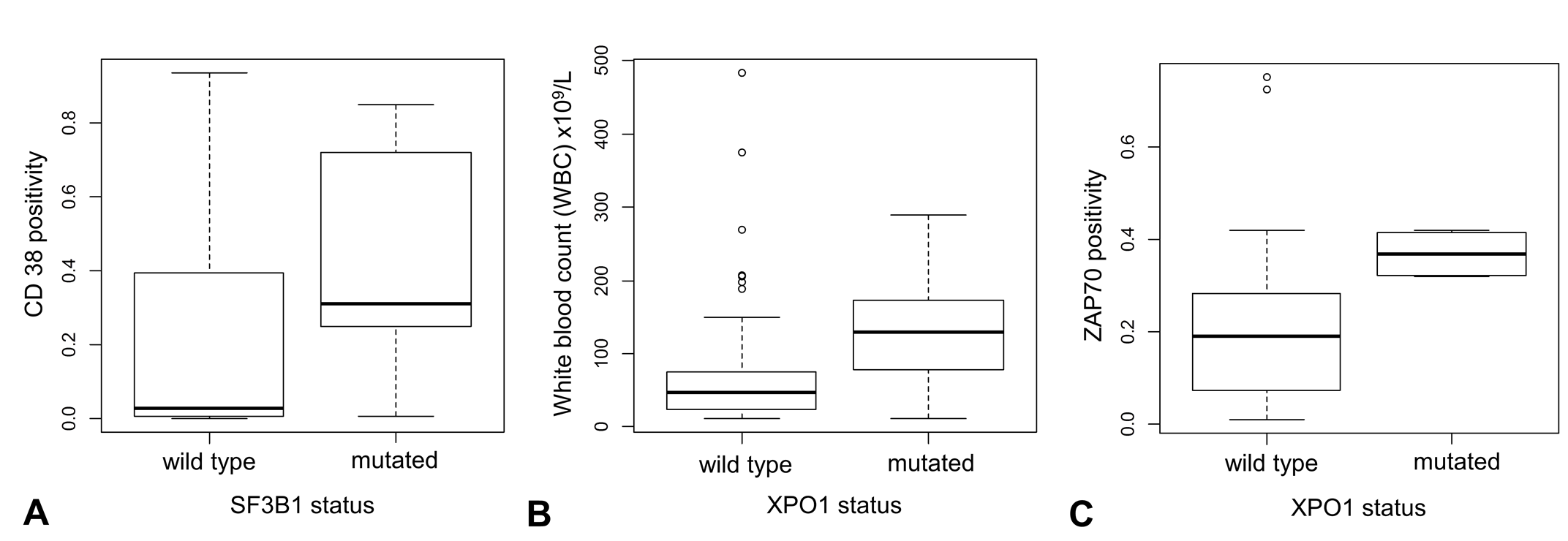

Supplement: S4 Fig — A) SF3B1 mutated untreated patients showed an increased CD38 expression (p<0.04). B and C) Patients harboring XPO1 mutations showed an increased WBC (p<0.001) and treated patients presented a higher ZAP70 expression compared to their wild type counterparts (p<0.03). (TIF) [file pone.0129544.s004.tif]
