# Supplementary material for: Comprehensive Analysis of Disease-Related Genes in Chronic Lymphocytic Leukemia by Multiplex PCR-Based Next Generation Sequencing
Source: PLoS One. 2015 Jun 8;10(6):e0129544. doi: 10.1371/journal.pone.0129544 (PMC4459702; doi:10.1371/journal.pone.0129544)
Supplement: S1 Table — (DOCX) [file pone.0129544.s005.docx]

S1 Table. Target regions of the CLL panel 1 and 2

| **Chromosome** | **Start** | **Stop** | **AmpliconID_Gene_Exon** |
| --- | --- | --- | --- |
| **Panel 1** | | | |
| chr1 | 9784025 | 9784137 | AMPL790398636_PIK3CD_Exon21# |
| chr1 | 9784136 | 9784275 | AMPL790401743_PIK3CD_Exon21# |
| chr1 | 9784294 | 9784427 | AMPL789461861_PIK3CD_Exon22# |
| chr1 | 9784427 | 9784605 | AMPL789494496_PIK3CD_Exon22# |
| chr1 | 9784840 | 9785017 | AMPL789532005_PIK3CD_Exon23# |
| chr1 | 9786876 | 9787019 | AMPL1023269090_PIK3CD_Exon24# |
| chr1 | 9787019 | 9787160 | AMPL965242047_PIK3CD_Exon24# |
| chr2 | 61719324 | 61719485 | AMPL604752115_XPO1_Exon15# |
| chr2 | 61719483 | 61719592 | AMPL586208437_XPO1_Exon15# |
| chr2 | 61719586 | 61719726 | AMPL631468458_XPO1_Exon15# |
| chr2 | 61719927 | 61720095 | AMPL867845130_XPO1_Exon13# |
| chr2 | 61720123 | 61720278 | AMPL631330509_XPO1_Exon13# |
| chr2 | 61720986 | 61721129 | AMPL586209492_XPO1_Exon12# |
| chr2 | 61721126 | 61721268 | AMPL586224083_XPO1_Exon12# |
| chr2 | 198266439 | 198266537 | AMPL529499302_SF3B1_Exon16# |
| chr2 | 198266522 | 198266691 | AMPL529502820_SF3B1_Exon16# |
| chr2 | 198266719 | 198266860 | AMPL529499628_SF3B1_Exon15# |
| chr2 | 198267149 | 198267321 | AMPL540707664_SF3B1_Exon14# |
| chr2 | 198267321 | 198267421 | AMPL529510076_SF3B1_Exon14# |
| chr2 | 198267421 | 198267556 | AMPL529523970_SF3B1_Exon14# |
| chr3 | 38182190 | 38182374 | AMPL461708423_MYD88_Exon4# |
| chr3 | 38182550 | 38182651 | AMPL461320681_MYD88_Exon5# |
| chr3 | 38182651 | 38182774 | AMPL461335271_MYD88_Exon5# |
| chr3 | 178928117 | 178928286 | AMPL391663014_PIK3CA_Exon9# |
| chr3 | 178928286 | 178928456 | AMPL391678526_PIK3CA_Exon9# |
| chr3 | 178935835 | 178936001 | AMPL431898893_PIK3CA_Exon10# |
| chr3 | 178936022 | 178936106 | AMPL393459515_PIK3CA_Exon10# |
| chr3 | 178936092 | 178936180 | AMPL766334235_PIK3CA_Exon10# |
| chr3 | 178936892 | 178937043 | AMPL439014560_PIK3CA_Exon11# |
| chr3 | 178937043 | 178937114 | AMPL391540460_PIK3CA_Exon11# |
| chr3 | 178947889 | 178948058 | AMPL929426275_PIK3CA_Exon20# |
| chr3 | 178948056 | 178948184 | AMPL393716297_PIK3CA_Exon20# |
| chr3 | 178952028 | 178952205 | AMPL392251341_PIK3CA_Exon21# |
| chr4 | 153249276 | 153249418 | AMPL749428714_FBXW7_Exon9# |
| chr4 | 153249418 | 153249550 | AMPL411831344_FBXW7_Exon9# |
| chr4 | 153250711 | 153250857 | AMPL731873228_FBXW7_Exon8# |
| chr4 | 153250857 | 153250992 | AMPL410695952_FBXW7_Exon8# |
| chr4 | 153251860 | 153252032 | AMPL687137193_FBXW7_Exon7# |
| chr4 | 153253709 | 153253847 | AMPL408308387_FBXW7_Exon6# |
| chr4 | 153253847 | 153254000 | AMPL422723675_FBXW7_Exon6# |
| chr9 | 139390107 | 139390280 | AMPL389071515_NOTCH1_Exon34# |
| chr9 | 139390278 | 139390410 | AMPL1023279207_NOTCH1_Exon34# |
| chr10 | 89692658 | 89692775 | AMPL703872780_PTEN_Exon5# |
| chr10 | 89692775 | 89692952 | AMPL703903618_PTEN_Exon5# |
| chr10 | 89711806 | 89711979 | AMPL968354751_PTEN_Exon6# |
| chr10 | 89711979 | 89712106 | AMPL623452574_PTEN_Exon6# |
| chr10 | 89724917 | 89725058 | AMPL703848673_PTEN_Exon9# |
| chr10 | 89725058 | 89725147 | AMPL391543592_PTEN_Exon9# |
| chr10 | 89725174 | 89725341 | AMPL391543631_PTEN_Exon9# |
| chr11 | 108173533 | 108173698 | AMPL637661621_ATM_Exon36# |
| chr11 | 108173698 | 108173792 | AMPL851616633_ATM_Exon36# |
| chr12 | 7067072 | 7067247 | AMPL789687311_PTPN6_Exon11# |
| chr12 | 7069032 | 7069212 | AMPL790061137_PTPN6_Exon12# |
| chr17 | 7576995 | 7577174 | AMPL705603720_TP53_Exon8# |
| chr17 | 7577356 | 7577509 | AMPL388733576_TP53_Exon7# |
| chr17 | 7577508 | 7577611 | AMPL387805586_TP53_Exon7# |
| chr17 | 7578150 | 7578333 | AMPL387845397_TP53_Exon6# |
| chr17 | 7578249 | 7578425 | AMPL387854314_TP53_Exon5# |
| chr17 | 7578425 | 7578555 | AMPL707393388_TP53_Exon5# |
| chr17 | 62006055 | 62006236 | AMPL755777037_CD79B_Exon6# |
| chr17 | 62006765 | 62006930 | AMPL551731808_CD79B_Exon5# |
| chr22 | 22127125 | 22127297 | AMPL527206685_MAPK1_Exon7# |
| chrX | 41202453 | 41202597 | AMPL801574439_DDX3X_Exon7# |
| chrX | 41202947 | 41203112 | AMPL801449024_DDX3X_Exon8# |
| chrX | 41203250 | 41203371 | AMPL801524167_DDX3X_Exon9# |
| chrX | 41204387 | 41204468 | AMPL801406997_DDX3X_Exon11# |
| chrX | 41204468 | 41204644 | AMPL801418667_DDX3X_Exon11# |
| chrX | 41205740 | 41205913 | AMPL801337081_DDX3X_Exon14# |
| chrX | 100609563 | 100609737 | AMPL551294195_BTK_Exon16# |
| chrX | 100610912 | 100611086 | AMPL552372528_BTK_Exon15# |
| chrX | 100611086 | 100611256 | AMPL552429753_BTK_Exon15# |
| chrX | 100611641 | 100611795 | AMPL611084046_BTK_Exon14# |
| chrX | 100611795 | 100611946 | AMPL551583129_BTK_Exon14# |
| **Panel 2** | | | |
| chr2 | 198256985 | 198257139 | AMPL529513592_SF3B1_Exon25# |
| chr2 | 198257139 | 198257306 | AMPL562020001_SF3B1_Exon25# |
| chr2 | 198257715 | 198257825 | AMPL3454705572_SF3B1_Exon24# |
| chr2 | 198257825 | 198257984 | AMPL3503992962_SF3B1_Exon24# |
| chr2 | 198260663 | 198260809 | AMPL576642473_SF3B1_Exon23# |
| chr2 | 198260809 | 198260936 | AMPL529499472_SF3B1_Exon23# |
| chr2 | 198260936 | 198261057 | AMPL529510434_SF3B1_Exon23# |
| chr2 | 198262638 | 198262809 | AMPL529510759_SF3B1_Exon22# |
| chr2 | 198262809 | 198262964 | AMPL587295092_SF3B1_Exon22# |
| chr2 | 198263136 | 198263280 | AMPL3673302042_SF3B1_Exon21# |
| chr2 | 198263278 | 198263399 | AMPL576333997_SF3B1_Exon21# |
| chr2 | 198264644 | 198264775 | AMPL604500653_SF3B1_Intron20# |
| chr2 | 198264775 | 198264937 | AMPL587280519_SF3B1_Exon20# |
| chr2 | 198264832 | 198265012 | AMPL604524655_SF3B1_Exon20#_Exon19# |
| chr2 | 198265012 | 198265184 | AMPL2758507014_SF3B1_Exon19# |
| chr2 | 198265322 | 198265491 | AMPL576642062_SF3B1_Exon18# |
| chr2 | 198265466 | 198265565 | AMPL529512492_SF3B1_Exon18# |
| chr2 | 198265565 | 198265681 | AMPL529515581_SF3B1_Exon18# |
| chr2 | 198266092 | 198266205 | AMPL555912744_SF3B1_Exon17# |
| chr2 | 198266178 | 198266278 | AMPL529500194_SF3B1_Exon17# |
| chr2 | 198266443 | 198266526 | AMPL1413798090_SF3B1_Exon16# |
| chr2 | 198266522 | 198266691 | AMPL529502820_SF3B1_Exon16# |
| chr2 | 198266575 | 198266719 | AMPL529503894_SF3B1_Exon16#_Exon15# |
| chr2 | 198267149 | 198267321 | AMPL540707664_SF3B1_Exon14# |
| chr2 | 198267321 | 198267421 | AMPL529510076_SF3B1_Exon14# |
| chr2 | 198267421 | 198267556 | AMPL529523970_SF3B1_Exon14# |
| chr2 | 198267590 | 198267708 | AMPL723505021_SF3B1_Exon13# |
| chr2 | 198267708 | 198267862 | AMPL556015216_SF3B1_Exon13# |
| chr2 | 198268218 | 198268391 | AMPL529521989_SF3B1_Exon12# |
| chr2 | 198268391 | 198268493 | AMPL529531715_SF3B1_Exon12# |
| chr2 | 198269791 | 198269860 | AMPL1407830555_SF3B1_Exon11# |
| chr2 | 198269822 | 198269979 | AMPL529515562_SF3B1_Exon11# |
| chr2 | 198270069 | 198270162 | AMPL529500017_SF3B1_Exon10# |
| chr2 | 198270162 | 198270303 | AMPL576519838_SF3B1_Exon10# |
| chr2 | 198272616 | 198272785 | AMPL3628433904_SF3B1_Exon9# |
| chr2 | 198272785 | 198272954 | AMPL3504129708_SF3B1_Exon9# |
| chr2 | 198273036 | 198273138 | AMPL1652590115_SF3B1_Exon8# |
| chr2 | 198273133 | 198273239 | AMPL3673265777_SF3B1_Exon8# |
| chr2 | 198273238 | 198273312 | AMPL3673283970_SF3B1_Exon8# |
| chr2 | 198274486 | 198274636 | AMPL529516469_SF3B1_Exon7# |
| chr2 | 198274634 | 198274752 | AMPL529527985_SF3B1_Exon7# |
| chr2 | 198281477 | 198281607 | AMPL529514659_SF3B1_Exon6# |
| chr2 | 198281607 | 198281679 | AMPL529525116_SF3B1_Exon6# |
| chr2 | 198283221 | 198283357 | AMPL529502653_SF3B1_Exon5# |
| chr2 | 198283577 | 198283740 | AMPL529501161_SF3B1_Exon4# |
| chr2 | 198285008 | 198285164 | AMPL723964157_SF3B1_Exon4# |
| chr2 | 198285164 | 198285332 | AMPL529528213_SF3B1_Exon4# |
| chr2 | 198285709 | 198285814 | AMPL529499999_SF3B1_Exon3# |
| chr2 | 198285814 | 198285948 | AMPL529503419_SF3B1_Exon3# |
| chr2 | 198288382 | 198288555 | AMPL723166508_SF3B1_Exon2# |
| chr2 | 198288555 | 198288728 | AMPL529524655_SF3B1_Exon2# |
| chr2 | 198299613 | 198299790 | AMPL529521539_SF3B1_Exon1# |
| chr3 | 38180094 | 38180255 | AMPL460903538_MYD88_Exon1# |
| chr3 | 38180243 | 38180382 | AMPL460906876_MYD88_Exon1# |
| chr3 | 38180382 | 38180555 | AMPL460925384_MYD88_Exon1# |
| chr3 | 38181339 | 38181514 | AMPL461139916_MYD88_Exon2# |
| chr3 | 38181785 | 38181959 | AMPL1186414652_MYD88_Exon3# |
| chr3 | 38181959 | 38182137 | AMPL1184868952_MYD88_Exon3# |
| chr3 | 38182190 | 38182374 | AMPL461708423_MYD88_Exon4# |
| chr3 | 38182550 | 38182651 | AMPL461320681_MYD88_Exon5# |
| chr3 | 38182651 | 38182805 | AMPL461335314_MYD88_Exon5# |
| chr9 | 139390444 | 139390610 | AMPL393483900_NOTCH1_Exon34# |
| chr9 | 139390609 | 139390742 | AMPL393490709_NOTCH1_Exon34# |
| chr9 | 139390724 | 139390880 | AMPL1733131768_NOTCH1_Exon34# |
| chr9 | 139390868 | 139391002 | AMPL687198331_NOTCH1_Exon34# |
| chr9 | 139390991 | 139391125 | AMPL393548366_NOTCH1_Exon34# |
| chr9 | 139391115 | 139391275 | AMPL393560912_NOTCH1_Exon34# |
| chr9 | 139391275 | 139391446 | AMPL393565691_NOTCH1_Exon34# |
| chr9 | 139391397 | 139391575 | AMPL393570760_NOTCH1_Exon34# |
| chr9 | 139391574 | 139391740 | AMPL393579722_NOTCH1_Exon34# |
| chr9 | 139391734 | 139391922 | AMPL393587649_NOTCH1_Exon34# |
| chr9 | 139391919 | 139392066 | AMPL393593091_NOTCH1_Exon34# |
| chr9 | 139393302 | 139393490 | AMPL1133733423_NOTCH1_Exon33# |
| chr9 | 139393467 | 139393591 | AMPL395255812_NOTCH1_Exon32# |
| chr9 | 139393575 | 139393746 | AMPL393462275_NOTCH1_Exon32# |
| chr9 | 139394896 | 139395014 | AMPL393550305_NOTCH1_Exon31# |
| chr9 | 139395014 | 139395193 | AMPL393552977_NOTCH1_Exon31# |
| chr9 | 139395176 | 139395311 | AMPL393558073_NOTCH1_Exon31# |
| chr9 | 139396066 | 139396226 | AMPL395395395_NOTCH1_Exon30# |
| chr9 | 139396224 | 139396370 | AMPL393876564_NOTCH1_Exon30# |
| chr9 | 139396395 | 139396568 | AMPL393468476_NOTCH1_Exon29# |
| chr9 | 139396665 | 139396782 | AMPL394076136_NOTCH1_Exon28# |
| chr9 | 139396782 | 139396963 | AMPL389056250_NOTCH1_Exon28# |
| chr9 | 139397611 | 139397796 | AMPL1406509564_NOTCH1_Exon27# |
| chr9 | 139399124 | 139399292 | AMPL3659581187_NOTCH1_Exon26# |
| chr9 | 139399289 | 139399458 | AMPL3659582003_NOTCH1_Exon26# |
| chr9 | 139399455 | 139399547 | AMPL389057328_NOTCH1_Exon26# |
| chr9 | 139399546 | 139399674 | AMPL576385984_NOTCH1_Exon26# |
| chr9 | 139399726 | 139399884 | AMPL393813581_NOTCH1_Exon25# |
| chr9 | 139399884 | 139400065 | AMPL3317928598_NOTCH1_Exon25# |
| chr9 | 139400065 | 139400193 | AMPL1489840519_NOTCH1_Exon25# |
| chr9 | 139400159 | 139400339 | AMPL393827630_NOTCH1_Exon25# |
| chr9 | 139400930 | 139401112 | AMPL393523557_NOTCH1_Exon24# |
| chr9 | 139401115 | 139401274 | AMPL393762385_NOTCH1_Exon23# |
| chr9 | 139401270 | 139401438 | AMPL393768721_NOTCH1_Exon23# |
| chr9 | 139401722 | 139401904 | AMPL974297171_NOTCH1_Exon22# |
| chr9 | 139402300 | 139402478 | AMPL395582111_NOTCH1_Exon21# |
| chr9 | 139402478 | 139402631 | AMPL3659630892_NOTCH1_Exon21# |
| chr9 | 139402655 | 139402843 | AMPL393826115_NOTCH1_Exon20# |
| chr9 | 139403260 | 139403426 | AMPL393847611_NOTCH1_Exon19# |
| chr9 | 139403426 | 139403608 | AMPL393851212_NOTCH1_Exon19# |
| chr9 | 139404133 | 139404315 | AMPL393835150_NOTCH1_Exon18# |
| chr9 | 139404315 | 139404423 | AMPL393843865_NOTCH1_Exon18# |
| chr9 | 139405071 | 139405234 | AMPL393483551_NOTCH1_Exon17# |
| chr9 | 139405234 | 139405392 | AMPL3318173741_NOTCH1_Exon17# |
| chr9 | 139405558 | 139405647 | AMPL3659461802_NOTCH1_Exon16# |
| chr9 | 139405645 | 139405763 | AMPL393716240_NOTCH1_Exon16# |
| chr9 | 139407433 | 139407620 | AMPL393674542_NOTCH1_Exon15# |
| chr9 | 139407826 | 139408005 | AMPL393475135_NOTCH1_Exon14# |
| chr9 | 139408878 | 139409020 | AMPL393872773_NOTCH1_Exon13# |
| chr9 | 139409019 | 139409203 | AMPL393887068_NOTCH1_Exon13# |
| chr9 | 139409696 | 139409872 | AMPL2686674775_NOTCH1_Exon12# |
| chr9 | 139409884 | 139410024 | AMPL394049893_NOTCH1_Exon11# |
| chr9 | 139410024 | 139410192 | AMPL394052386_NOTCH1_Exon11# |
| chr9 | 139410300 | 139410474 | AMPL1838015592_NOTCH1_Exon10# |
| chr9 | 139410471 | 139410589 | AMPL393840759_NOTCH1_Exon10# |
| chr9 | 139411745 | 139411898 | AMPL395901075_NOTCH1_Exon9# |
| chr9 | 139412137 | 139412280 | AMPL393459313_NOTCH1_Exon8# |
| chr9 | 139412275 | 139412438 | AMPL393467389_NOTCH1_Exon8# |
| chr9 | 139412495 | 139412680 | AMPL3299434711_NOTCH1_Exon7# |
| chr9 | 139412679 | 139412864 | AMPL396036289_NOTCH1_Exon7# |
| chr9 | 139412951 | 139413132 | AMPL393577583_NOTCH1_Exon6# |
| chr9 | 139413132 | 139413282 | AMPL3386297428_NOTCH1_Exon6# |
| chr9 | 139413836 | 139413982 | AMPL1406505740_NOTCH1_Exon5# |
| chr9 | 139413981 | 139414083 | AMPL1406505750_NOTCH1_Exon5# |
| chr9 | 139417188 | 139417356 | AMPL3299973779_NOTCH1_Exon4# |
| chr9 | 139417311 | 139417501 | AMPL393534998_NOTCH1_Exon4# |
| chr9 | 139417501 | 139417645 | AMPL393537721_NOTCH1_Exon4# |
| chr9 | 139418097 | 139418203 | AMPL393677786_NOTCH1_Exon3# |
| chr9 | 139418201 | 139418337 | AMPL393683751_NOTCH1_Exon3# |
| chr9 | 139418335 | 139418441 | AMPL393687962_NOTCH1_Exon3# |
| chr9 | 139438446 | 139438603 | AMPL393839516_NOTCH1_Exon2# |
| chr11 | 108098340 | 108098500 | AMPL392261218_ATM_Exon2# |
| chr11 | 108098395 | 108098552 | AMPL391997777_ATM_Exon2#_Exon#3 |
| chr11 | 108098552 | 108098686 | AMPL623973469_ATM_Exon3# |
| chr11 | 108099804 | 108099959 | AMPL391948634_ATM_Exon4# |
| chr11 | 108099959 | 108100072 | AMPL391991577_ATM_Exon4# |
| chr11 | 108106358 | 108106525 | AMPL391522368_ATM_Exon5# |
| chr11 | 108106505 | 108106603 | AMPL391536984_ATM_Exon5# |
| chr11 | 108114587 | 108114757 | AMPL447455904_ATM_Exon6# |
| chr11 | 108114728 | 108114818 | AMPL3660815253_ATM_Exon6# |
| chr11 | 108114808 | 108114959 | AMPL409275345_ATM_Exon6# |
| chr11 | 108115454 | 108115549 | AMPL391579143_ATM_Exon7# |
| chr11 | 108115549 | 108115700 | AMPL391589757_ATM_Exon7# |
| chr11 | 108115658 | 108115784 | AMPL411498022_ATM_Exon7# |
| chr11 | 108117627 | 108117790 | AMPL1482570325_ATM_Exon8# |
| chr11 | 108117717 | 108117865 | AMPL699213618_ATM_Exon8# |
| chr11 | 108119695 | 108119765 | AMPL391558752_ATM_Exon9# |
| chr11 | 108119765 | 108119883 | AMPL624222416_ATM_Exon9# |
| chr11 | 108121359 | 108121498 | AMPL623995564_ATM_Exon10# |
| chr11 | 108121498 | 108121589 | AMPL392330115_ATM_Exon10# |
| chr11 | 108121589 | 108121758 | AMPL3660799045_ATM_Exon10# |
| chr11 | 108121758 | 108121911 | AMPL3431977515_ATM_Exon10# |
| chr11 | 108122552 | 108122622 | AMPL1549898614_ATM_Exon11# |
| chr11 | 108122622 | 108122789 | AMPL1482037524_ATM_Exon11# |
| chr11 | 108123527 | 108123694 | AMPL392071108_ATM_Exon12# |
| chr11 | 108124511 | 108124639 | AMPL391670871_ATM_Exon13# |
| chr11 | 108124639 | 108124805 | AMPL687292966_ATM_Exon13# |
| chr11 | 108126829 | 108126991 | AMPL391466017_ATM_Exon14# |
| chr11 | 108126989 | 108127160 | AMPL437230929_ATM_Exon14# |
| chr11 | 108128145 | 108128253 | AMPL1186328350_ATM_Exon15# |
| chr11 | 108128247 | 108128318 | AMPL391971013_ATM_Exon15# |
| chr11 | 108129604 | 108129757 | AMPL391704487_ATM_Exon16# |
| chr11 | 108129757 | 108129913 | AMPL391707792_ATM_Exon16# |
| chr11 | 108137856 | 108137954 | AMPL3660734218_ATM_Exon17# |
| chr11 | 108137953 | 108138110 | AMPL392298588_ATM_Exon17# |
| chr11 | 108139103 | 108139224 | AMPL3660785676_ATM_Exon18# |
| chr11 | 108139224 | 108139372 | AMPL3660795306_ATM_Exon18# |
| chr11 | 108141801 | 108141965 | AMPL391926752_ATM_Exon19# |
| chr11 | 108141821 | 108141993 | AMPL394748692_ATM_Exon19#_Exon#20# |
| chr11 | 108143142 | 108143309 | AMPL405925555_ATM_Exon21# |
| chr11 | 108143283 | 108143417 | AMPL1186569991_ATM_Exon21# |
| chr11 | 108143454 | 108143598 | AMPL3660734964_ATM_Exon22# |
| chr11 | 108150159 | 108150253 | AMPL394889034_ATM_Exon23# |
| chr11 | 108150253 | 108150428 | AMPL437719184_ATM_Exon23# |
| chr11 | 108151630 | 108151799 | AMPL622791648_ATM_Exon24# |
| chr11 | 108151799 | 108151957 | AMPL391714171_ATM_Exon24# |
| chr11 | 108153428 | 108153506 | AMPL3660881930_ATM_Exon25# |
| chr11 | 108153499 | 108153674 | AMPL394898140_ATM_Exon25# |
| chr11 | 108154887 | 108154996 | AMPL392039731_ATM_Exon26# |
| chr11 | 108154993 | 108155128 | AMPL1493143374_ATM_Exon26# |
| chr11 | 108155125 | 108155205 | AMPL3660806661_ATM_Exon26# |
| chr11 | 108158173 | 108158344 | AMPL398028992_ATM_Exon27# |
| chr11 | 108158344 | 108158491 | AMPL3660799178_ATM_Exon27# |
| chr11 | 108159655 | 108159818 | AMPL394737832_ATM_Exon28# |
| chr11 | 108159759 | 108159838 | AMPL410139130_ATM_Exon28# |
| chr11 | 108160294 | 108160451 | AMPL1484991368_ATM_Exon29# |
| chr11 | 108160381 | 108160553 | AMPL391647032_ATM_Exon29# |
| chr11 | 108163272 | 108163437 | AMPL624516207_ATM_Exon30# |
| chr11 | 108163434 | 108163563 | AMPL396418276_ATM_Exon30# |
| chr11 | 108164008 | 108164168 | AMPL424101530_ATM_Exon31# |
| chr11 | 108164140 | 108164253 | AMPL411271719_ATM_Exon31# |
| chr11 | 108165630 | 108165801 | AMPL391523578_ATM_Exon32# |
| chr11 | 108167987 | 108168078 | AMPL1034497409_ATM_Exon33# |
| chr11 | 108170412 | 108170552 | AMPL548404054_ATM_Exon34# |
| chr11 | 108170537 | 108170648 | AMPL3660881219_ATM_Exon34# |
| chr11 | 108172329 | 108172409 | AMPL392000436_ATM_Exon35# |
| chr11 | 108172409 | 108172542 | AMPL886331227_ATM_Exon35# |
| chr11 | 108173541 | 108173697 | AMPL3660816598_ATM_Exon36# |
| chr11 | 108173697 | 108173792 | AMPL3660816898_ATM_Exon36# |
| chr11 | 108175384 | 108175481 | AMPL391465960_ATM_Exon37# |
| chr11 | 108175446 | 108175525 | AMPL391468297_ATM_Exon37# |
| chr11 | 108175525 | 108175638 | AMPL564204458_ATM_Exon37# |
| chr11 | 108178586 | 108178747 | AMPL3660792931_ATM_Exon38# |
| chr11 | 108180811 | 108180961 | AMPL401725247_ATM_Exon39# |
| chr11 | 108180961 | 108181074 | AMPL391988106_ATM_Exon39# |
| chr11 | 108182987 | 108183139 | AMPL1105767645_ATM_Exon40# |
| chr11 | 108183139 | 108183282 | AMPL391653694_ATM_Exon40# |
| chr11 | 108186543 | 108186680 | AMPL391887107_ATM_Exon41# |
| chr11 | 108186710 | 108186875 | AMPL391557630_ATM_Exon42# |
| chr11 | 108188069 | 108188163 | AMPL403099818_ATM_Exon43# |
| chr11 | 108188144 | 108188275 | AMPL392139446_ATM_Exon43# |
| chr11 | 108190618 | 108190692 | AMPL3660792146_ATM_Exon44# |
| chr11 | 108190692 | 108190824 | AMPL392158627_ATM_Exon44# |
| chr11 | 108191947 | 108192064 | AMPL391469365_ATM_Exon45# |
| chr11 | 108192064 | 108192167 | AMPL391477050_ATM_Exon45# |
| chr11 | 108196027 | 108196166 | AMPL392048296_ATM_Exon46# |
| chr11 | 108196166 | 108196330 | AMPL392159002_ATM_Exon46# |
| chr11 | 108196788 | 108196881 | AMPL395528661_ATM_Exon47# |
| chr11 | 108196802 | 108196889 | AMPL392242365_ATM_Exon47# |
| chr11 | 108198241 | 108198405 | AMPL433902805_ATM_Exon48# |
| chr11 | 108198405 | 108198495 | AMPL391740586_ATM_Exon48# |
| chr11 | 108199749 | 108199822 | AMPL1706343703_ATM_Exon49# |
| chr11 | 108199817 | 108199906 | AMPL391485841_ATM_Exon49# |
| chr11 | 108199906 | 108200019 | AMPL391496996_ATM_Exon49# |
| chr11 | 108200917 | 108201010 | AMPL637532870_ATM_Exon50# |
| chr11 | 108201010 | 108201153 | AMPL3660813389_ATM_Exon50# |
| chr11 | 108202073 | 108202231 | AMPL391465932_ATM_Exon51# |
| chr11 | 108202231 | 108202400 | AMPL425276545_ATM_Exon51# |
| chr11 | 108202507 | 108202681 | AMPL391795564_ATM_Exon52# |
| chr11 | 108202681 | 108202847 | AMPL624574569_ATM_Exon52# |
| chr11 | 108203399 | 108203517 | AMPL623313100_ATM_Exon53# |
| chr11 | 108203517 | 108203651 | AMPL391664576_ATM_Exon53# |
| chr11 | 108204595 | 108204673 | AMPL391502501_ATM_Exon54# |
| chr11 | 108204689 | 108204827 | AMPL426749282_ATM_Exon54# |
| chr11 | 108205569 | 108205735 | AMPL410139362_ATM_Exon55# |
| chr11 | 108205732 | 108205847 | AMPL391694137_ATM_Exon55# |
| chr11 | 108206500 | 108206621 | AMPL391771149_ATM_Exon56# |
| chr11 | 108206621 | 108206697 | AMPL651267058_ATM_Exon56# |
| chr11 | 108213815 | 108213971 | AMPL397937009_ATM_Exon57# |
| chr11 | 108213971 | 108214138 | AMPL391562624_ATM_Exon57# |
| chr11 | 108216525 | 108216652 | AMPL3660877791_ATM_Exon58# |
| chr11 | 108217986 | 108218131 | AMPL1035082608_ATM_Exon59# |
| chr11 | 108224475 | 108224641 | AMPL3660764478_ATM_Exon60# |
| chr11 | 108225506 | 108225661 | AMPL3660740856_ATM_Exon61# |
| chr11 | 108235661 | 108235830 | AMPL1312142110_ATM_Exon62# |
| chr11 | 108235830 | 108235957 | AMPL1503005649_ATM_Exon62# |
| chr11 | 108235983 | 108236158 | AMPL1035106763_ATM_Exon63# |
| chr11 | 108236158 | 108236296 | AMPL391548043_ATM_Exon63# |
| chr17 | 7572847 | 7573025 | AMPL388592559_TP53_Exon11# |
| chr17 | 7573863 | 7574041 | AMPL387894777_TP53_Exon10# |
| chr17 | 7576424 | 7576537 | AMPL4296982438_TP53_Exon9# |
| chr17 | 7576566 | 7576697 | AMPL4297343428_TP53_Exon9# |
| chr17 | 7576789 | 7576968 | AMPL1514447650_TP53_Exon9# |
| chr17 | 7576995 | 7577174 | AMPL705603720_TP53_Exon8# |
| chr17 | 7577356 | 7577509 | AMPL388733576_TP53_Exon7# |
| chr17 | 7577508 | 7577613 | AMPL387805648_TP53_Exon7# |
| chr17 | 7578150 | 7578333 | AMPL387845397_TP53_Exon6# |
| chr17 | 7578249 | 7578425 | AMPL2449945349_TP53_Exon6#_Exon7# |
| chr17 | 7578425 | 7578560 | AMPL707393441_TP53_Exon5# |
| chr17 | 7579234 | 7579385 | AMPL624460718_TP53_Exon4# |
| chr17 | 7579385 | 7579522 | AMPL651471237_TP53_Exon4# |
| chr17 | 7579522 | 7579684 | AMPL388679445_TP53_Exon4# |
| chr17 | 7579609 | 7579766 | AMPL1547505015_TP53_Exon3# |
| chr17 | 7579782 | 7579964 | AMPL388009515_TP53_Exon2# |
