# Supplementary material for: Comprehensive Analysis of Disease-Related Genes in Chronic Lymphocytic Leukemia by Multiplex PCR-Based Next Generation Sequencing
Source: PLoS One. 2015 Jun 8;10(6):e0129544. doi: 10.1371/journal.pone.0129544 (PMC4459702; doi:10.1371/journal.pone.0129544)
Supplement: S2 Table — DNA quantification was done using native DNA from HEK-293 (human embryonic kidney) cells without known gene mutations; all samples were measured in duplicates. (DOCX) [file pone.0129544.s006.docx]

S2 Table: Parameters for the *HFE* qPRC. DNA quantification was done using native DNA from HEK-293 (human embryonic kidney) cells without known gene mutations; all samples were measured in duplicates.

| **A) *HFE* qPCR Setup** | | | |
| --- | --- | --- | --- |
| **Component** | | | **Volume [µl]** |
| Nuclease-free water | | | 7.4 |
| *HFE* primer forward (10 µM)*^1^ | | | 0.8 |
| *HFE* primer reverse (10 µM)*^2^ | | | 0.8 |
| GoTaq qPCR Master Mix (Promega) | | | 10.0 |
| Total | | | 19.0 |
| gDNA (10 ng) | | | 1.0 |
| **Total** | | | **20.0** |
| *^1^5’ATG GAT GCC AAG GAG TTC GAA CC | | | |
| *^2^ 5’GCC ATA ATT ACC TCC TCA GGC AC | | | |
| **B) *HFE* qPCR Amplification Parameters** | | | |
| **Stage** | **Temperature** | **Time** | |
| Hold | 94°C | 3 minutes | |
| 55 cycles | 94°C | 30 seconds | |
|  | 60°C | 30 seconds | |
|  | 72°C | 30 seconds | |
| Plate read and melting curve | | | |
