# Supplementary material for: Comprehensive Analysis of Disease-Related Genes in Chronic Lymphocytic Leukemia by Multiplex PCR-Based Next Generation Sequencing
Source: PLoS One. 2015 Jun 8;10(6):e0129544. doi: 10.1371/journal.pone.0129544 (PMC4459702; doi:10.1371/journal.pone.0129544)
Supplement: S3 Table — A) PCR components; B) PCR conditions. Amplicon library quantification was performed with 5-fold dilutions of PhiX Control V3 (Illumina, San Diego, CA, USA) in a range from 0.064 up to 40 pM as reference standard. The library samples were diluted 1:4000 and measured in duplicates. (DOCX) [file pone.0129544.s007.docx]

S3 Table. PCR parameters for quantification of the constructed libraries by qPCR: A) PCR components and B) PCR conditions. Amplicon library quantification was performed with 5-fold dilutions of PhiX Control V3 (Illumina, San Diego, CA, USA) in a range from 0.064 up to 40 pM as reference standard. The library samples were diluted 1:4000 and measured in duplicates.

| **A) qPCR Setup** | | | |
| --- | --- | --- | --- |
| **Component** | | **Volume [µl]** | |
| Nuclease-free water | | 7.4 | |
| Forward primer (10 µM)*^1^ | | 0.8 | |
| Reverse primer (10 µM)*^2^ | | 0.8 | |
| GoTaq qPCR Master Mix (Promega) | | 10.0 | |
| Total | | 19.0 | |
| DNA library (diluted 1:4,000) | | 1.0 | |
| **Total** | | **20.0** | |
| *^1^ 5’AAT GAT ACG GCG ACC ACC GAG ATC TAC AC | | | |
| *^2^ 5’CAA GCA GAA GAC GGC ATA CGA GAT | | | |
| **B) qPCR Amplification Parameters** | | | |
| **Stage** | **Temperature** | | **Time** |
| Hold | 94°C | | 3 minutes |
| 30 cycles | 94°C | | 30 seconds |
|  | 60°C | | 30 seconds |
|  | 72°C | | 30 seconds |
