# Supplementary material for: Comprehensive Analysis of Disease-Related Genes in Chronic Lymphocytic Leukemia by Multiplex PCR-Based Next Generation Sequencing
Source: PLoS One. 2015 Jun 8;10(6):e0129544. doi: 10.1371/journal.pone.0129544 (PMC4459702; doi:10.1371/journal.pone.0129544)
Supplement: S4 Table — (DOCX) [file pone.0129544.s008.docx]

S4 Table. Primer used for Sanger sequencing validation. chr. chromosome; bp base pairs; hg human genome

| **Target** | **Genomic Position (hg19)** | **Target Length** | **Sequence (5'->3')** |
| --- | --- | --- | --- |
| ATM Exon 3 | chr.11:108098525-108098716 | 192 bp | GCCTGATTCGAGATCCTGAAACAATTAA |
|  |  |  | GCAAAGATAAATGTTAAGACTTACACACAA |
| ATM Exon 5 | chr11:108106323-108106618 | 295 bp | TAAATAGTTGCCATTCCAAGTGTC |
|  |  |  | TGGTGAAGTTTCATTTCATGAGG |
| ATM Exon 6 | chr.11:108114558-108114987 | 271 bp | ATTAGTGCAGTTTTAAAATCCTTTTTCTGT |
|  |  |  | TCTCTTTAGGAATCCACTAGTTCTGTTA |
| ATM Exon 17 | chr.11:108137832-108138138 | 307 bp | AAATTTTGACTACAGCATGCTCCTG |
|  |  |  | GAGGCCTCTTATACTGCCAAATCAATAT |
| ATM Exon 22 | chr.11:108143432-108143623 | 192 bp | CAGTTCTTTTCCCGTAGGCTGAT |
|  |  |  | CATCTGCAGCATTCCAAATACTTCA |
| ATM Exon 35 | chr.11:108172301-108172571 | 271 bp | TTTTCAGTGGAGGTTAACATTCATCAAGA |
|  |  |  | ACAGAACTGTTTTAGATATGCTGGGTATT |
| ATM Exon 48 | chr.11:108198217-108198523 | 307 bp | ATAGTTGTATGGCAAAAGCAGATGA |
|  |  |  | CTAAGTAACTATCTTAAGGGTTGCTCCA |
| ATM Exon 56 | chr11:108206495-108206788 | 294 bp | TTGCTATTCTCAGATGACTCTGTG |
|  |  |  | GCCTCCCAAAGCATTATGAATATG |
| DDX3X Exon 9 | chr.X:41203225-41203398 | 174 bp | TGATGAACTTTTCAAACAGGGTAGGT |
|  |  |  | CTGGAACTCATACTTACTTTTCTGGCT |
| MYD88 Exon 3 | chr.3:38182940-38182156 | 217 bp | GCAGGAGATGATCCGGCAAC |
|  |  |  | GCTGGACAGTGCACAGCTA |
| MYD88 Exon 5 | chr.3:38182530-38182829 | 300 bp | GGGATGGCTGTTGTTAACCCT |
|  |  |  | GTACATGGACAGGCAGACAGATAC |
| NOTCH1 Exon 14 | chr.9:139407809-139408023 | 215 bp | CCTCCCTCGACCTGCAGT |
|  |  |  | GGCCCTCTGCACTGAGAA |
| NOTCH1Exon 16 | chr.9:139405541-139405783 | 243 bp | CCGATTTGGGAGATCCCTCT |
|  |  |  | CCTGTGTCCCGCAGACAT |
| NOTCH1 Exon 23 | chr.9:139401097-139401457 | 361 bp | GCTTGGGCCACTGACGAAA |
|  |  |  | GGTAAGAGCAGGGCAGTGA |
| NOTCH1 Exon 25 | chr.9:139399866-139400359 | 494bp | ACCGTCCTGTCTTCCCTCTC |
|  |  |  | CAGAGACTGCGTGCAGTTC |
| NOTCH1 Exon 34 | chr.9:139390592-139391023 | 432 bp | TTACAGATGCAGCAGCAGAAC |
|  |  |  | GGACCAGTCGGAGACGTT |
| PTPN6 Exon 11 | chr.12:7067054-7067272 | 219 bp | GGGCACTGACCCTATGTCC |
|  |  |  | AGCTGTCACTACTACCATCAGGATT |
| SF3B1 Exon 7 | chr.2:198274465-198274659 | 195 bp | GCAACCCCAGGCTCAAAAATATG |
|  |  |  | CCCACACACCCATACTCCACTA |
| SF3B1 Exon 14 | chr.2:198267128-198267397 | 457 bp | ACTCATGACTGTCCTTTCTTTGTTTACA |
|  |  |  | ACAGGCTGTGTGTGTACCTCTA |
| SF3B1 Exon 15 | chr.2:198266692-198266886 | 195 bp | GGCATAGTTAAAACCTGTGTTTGGTT |
|  |  |  | GTAATTGGTGGATTTACCTTTCCTCTGT |
| SF3B1 Exon 16 | chr.2:198266409-198266719 | 311 bp | ACAGAGGAAAGGTAAATCCACCAATTAC |
|  |  |  | ATTCTGTTAGAACCATGAAACATATCCAGTT |
| SF3B1 Exon 18 | chr.2:198265302-198265519 | 218 bp | GGAGCAGCAGATATTGATCATAAACTTG |
|  |  |  | TATCGTTTGGTAACCCCCTGA |
| TP53 Exon 5 | chr.17:7,578,228-7,578,579 | 352 bp | CTCTGTCTCCTTCCTCTTCCTACA |
|  |  |  | ATCCAAATACTCCACACGCAAA |
| TP53 Exon 7 | chr.17:7,577,489-7,577,634 | 146 bp | CATCTTGGGCCTGTGTTATCTCC |
|  |  |  | GGCTCCTGACCTGGAGTCTT |
| XPO1 Exon 15 | chr.2: 61719302-61719749 | 448 bp | GCAATGCATGAAGAGGACGAAAA |
|  |  |  | ACAAGCCATATCCTGGACTCCAT |
