# Supplementary material for: Comprehensive Analysis of Disease-Related Genes in Chronic Lymphocytic Leukemia by Multiplex PCR-Based Next Generation Sequencing
Source: PLoS One. 2015 Jun 8;10(6):e0129544. doi: 10.1371/journal.pone.0129544 (PMC4459702; doi:10.1371/journal.pone.0129544)
Supplement: S5 Table — A1 and 2) Components and conditions for amplification of target regions by PCR; B1 and 2) Components and conditions for Sanger sequencing reaction. (DOCX) [file pone.0129544.s009.docx]

S5 Table. PCR and reaction parameters for Sanger sequencing: A1 and 2) components and conditions for amplification of target regions by PCR; B1 and 2) components and conditions for Sanger sequencing of target regions.

| **A1) PCR Setup** | | | |  | **B1) Sanger Reaction Setup** | | | |
| --- | --- | --- | --- | --- | --- | --- | --- | --- |
| **Component** | | **Volume [µl]** | |  | **Component** | | | **Volume [µl]** |
| Nuclease-free water | | 11.5-Y | |  | Nuclease-free water | | | 13.5 |
| Primer forward (10 µM) | | 0.5 | |  | Primer forward or reverse (10 µM) | | | 0.5 |
| Primer reverse (10 µM) | | 0.5 | |  | 5x Big Dye sequencing buffer (Life Technologies) | | | 4.5 |
| Multiplex PCR Master Mix (Qiagen) | | 12.5 | |  | Big Dye Terminator v3.1 Cycle (Life Technologies) | | | 0.5 |
| Total | | 24.0 | |  | Total | | | 24.0 |
| gDNA (10 ng) | | Y | |  | DNA amplicons | | | 1.0 |
| **Total** | | **25.0** | |  | **Total** | | | **20.0** |
| **A2) PCR Amplification Parameters** | | | |  | **B2) Sanger Reaction Parameters** | | | |
| **Stage** | **Temperature** | | **Time** |  | **Stage** | **Temperature** | **Time** | |
| Hold | 94°C | | 15 min |  | Hold | 95°C | 1 min | |
| 40 cycles | 94°C | | 30 sec |  | 34cycles | 96°C | 30 sec | |
|  | X°C* | | 90 sec |  |  | X°C* | 60 sec | |
|  | 72°C | | 60 sec |  |  | 60°C | 4 min | |
| Hold | 72°C | | 10 min |  | Hold | 60°C | 10 min | |
| * Annealing temperature of specific primer pair | | | | | | | | |
