# Supplementary material for: Comprehensive Analysis of Disease-Related Genes in Chronic Lymphocytic Leukemia by Multiplex PCR-Based Next Generation Sequencing
Source: PLoS One. 2015 Jun 8;10(6):e0129544. doi: 10.1371/journal.pone.0129544 (PMC4459702; doi:10.1371/journal.pone.0129544)
Supplement: S7 Table — (DOCX) [file pone.0129544.s011.docx]

S7 Table. Run parameters from the five MiSeq sequencing runs

| **Parameters** | **Run 1** | **Run 2** | **Run 3** | **Run 4** | **Run 5** | **Average** |
| --- | --- | --- | --- | --- | --- | --- |
| **Entity** | CLL | CLL | CLL | CLL | CLL |  |
| **Samples** | 36 | 31 | 29 | 30 | 29 |  |
| **Amplicons [n]** | 338 | 338 | 338 | 338 | 338 |  |
| **Cluster Density [K/mm²]** | 192 | 590 | 1,277 | 1,382 | 1,380 | 964 |
| **Cluster PF** | 90.07% | 93.77% | 89.27% | 69.78% | 88.67% | 86.31% |
| **Reads** | 3.75 M | 11.85 M | 24.38 M | 23.76 M | 26.37 M | 18.02 M |
| **Reads PF** | 3.38 M | 11.11 M | 21.76 M | 17.23 M | 23.38 M | 15.37 M |
| **Total Yield** | 1.00 Gb | 3.4 Gb | 6.6 Gb | 5.3 Gb | 7.1 Gb | 4.7 Gb |
| **≥ Q30 Score** | 97.70% | 97.70% | 95.30% | 90.60% | 95.00% | 95.26% |
| PF passed filter; Q30 Score 0.1% chance of wrong base call; M million; Gb gigabases | | | | | | |
