# Supplementary material for: Comprehensive Analysis of Disease-Related Genes in Chronic Lymphocytic Leukemia by Multiplex PCR-Based Next Generation Sequencing
Source: PLoS One. 2015 Jun 8;10(6):e0129544. doi: 10.1371/journal.pone.0129544 (PMC4459702; doi:10.1371/journal.pone.0129544)
Supplement: S8 Table — Bold mutations were confirmed by Sanger sequencing. fs frame shift; * stop gained; Freq frequency; Cov coverage; dbSNP single nucleotide variants database. (DOCX) [file pone.0129544.s012.docx]

S8 Table. A complete list of non-synonymous mutations ≥ 5% allelic frequency; bold variants were confirmed by Sanger sequencing. chr chromosome; fs frame shift; *stop gained; Freq frequency; Cov coverage; dbSNP single nucleotide variants database

| **Gene Name** | **Start Position** | **Transcript** | **Variant Type** | **cDNA Change** | **Protein Change** | **Allelic Freq.** | **Cov.** | **Patient** | **dbSNP** |
| --- | --- | --- | --- | --- | --- | --- | --- | --- | --- |
| *^†^ATM* | chr11:108098417 | NM_000051 | fsdeletion | c.66delA | p.E22fs | 80% | 10,198 | P114 | - |
| *^†^****ATM*** | **chr11:108114703** | **NM_000051** | **deletion** | **c.520_525delCTCTAT** | **p.L174_Y175del** | **41%** | **137** | **P031** | **-** |
| *^§^ATM* | chr11:108115522 | NM_000051 | missense | c.670A>G | p.K224E | 54% | 1,070 | P134 | - |
| *^§^ATM* | chr11:108128253 | NM_000051 | missense | c.2296A>G | p.K766E | 8% | 307 | P085 | - |
| *^§^ATM* | chr11:108128253 | NM_000051 | missense | c.2296A>G | p.K766E | 55% | 369 | P093 | - |
| *^†^ATM* | chr11:108143293 | NM_000051 | missense | c.3112G>C | p.V1038L | 47% | 1,428 | P115 | - |
| *^§^****ATM*** | **chr11:108143470** | **NM_000051** | **missense** | **c.3175G>A** | **p.A1059T** | **48%** | **5,088** | **P119** | **-** |
| *^§^ATM* | chr11:108143552 | NM_000051 | missense | c.3257G>A | p.R1086H | 50% | 2,477 | P042 | - |
| ***^¥^ATM*** | **chr11:108172404** | **NM_000051** | **missense** | **c.5207G>A** | **p.C1736Y** | **66%** | **130** | **P122** | **-** |
| ***^¥^****ATM* | chr11:108173733 | NM_000051 | fsdeletion | c.5473delC | p.Q1825fs | 99% | 3,383 | P079 | - |
| *^†^ATM* | chr11:108186557 | NM_000051 | missense | c.6014T>C | p.L2005P | 6% | 3,100 | P114 | - |
| *^†^ATM* | chr11:108186610 | NM_000051 | missense | c.6067G>A | p.G2023R | 48% | 3,394 | P076 | - |
| *^§^ATM* | chr11:108205835 | NM_000051 | missense | c.8150A>C | p.K2717T | 8% | 414 | P058 | - |
| *^§^****ATM*** | **chr11:108206609** | **NM_000051** | **missense** | **c.8189A>G** | **p.Q2730R** | **45%** | **5,059** | **P105** | **-** |
| ***^¥^****ATM* | chr11.108216545 | NM_000051 | missense | c.8494C>T | p.R2832C | 7% | 2,831 | P048 | - |
| *^†^ATM* | chr11:108236203 | NM_000051 | nonsense | c.9139C>T | p.R3047* | 28% | 1,052 | P074 | - |
| *^†^****DDX3X*** | **chrX:41203340** | **NM_001356** | **missense** | **c.823A>C** | **p.T275P** | **96%** | **572** | **P119** | **-** |
| *^†^****MYD88*** | **chr3:38182025** | **NM_002468** | **missense** | **c.649G>T** | **p.V217F** | **44%** | **2,427** | **P103** | **-** |
| *^†^****MYD88*** | **chr3:38182641** | **NM_002468** | **missense** | **c.613T>C** | **p.L265P** | **34%** | **3,209** | **P078** | **-** |
| *^†^NOTCH1* | chr9:139413193 | NM_017617 | missense | c.949G>A | p.G317S | 48% | 6,738 | P074 | - |
| *^†^NOTCH1* | chr9:139413163 | NM_017617 | missense | c.979T>C | p.W327R | 5% | 4,765 | P061 | - |
| *^†^NOTCH1* | chr9:139410544 | NM_017617 | missense | c.1558T>C | p.F520L | 7% | 104 | P134 | - |
| *^†^NOTCH1* | chr9:139407559 | NM_017617 | missense | c.2381A>G | p.E794G | 8% | 148 | P039 | - |
| *^†^NOTCH1* | chr9:139407559 | NM_017617 | missense | c.2381A>G | p.E794G | 7% | 155 | P093 | - |
| *^†^NOTCH1* | chr9:139405111 | NM_017617 | missense | c.2734C>T | p.R912W | 40% | 45 | P097 | - |
| *^†^NOTCH1* | chr9:139399327 | NM_017617 | missense | c.4816T>C | p.F1606L | 5% | 166 | P046 | - |
| *^†^NOTCH1* | chr9:139391908 | NM_017617 | missense | c.6283C>T | p.R2095C | 52% | 651 | P094 | - |
| *^†^****NOTCH1*** | **chr9:139390945** | **NM_017617** | **nonsense** | **c.7246C>T** | **p.Q2416*** | **62%** | **165** | **P028** | **-** |
| *^†^****NOTCH1*** | **chr9:139390813** | **NM_017617** | **nonsense** | **c.7378G>T** | **p.E2460*** | **25%** | **2,511** | **P072** | **-** |
| *^†^****NOTCH1*** | **chr9:139390649** | **NM_017617** | **fsdeletion** | **c.7541_7542delCT** | **p.P2514fs** | **29%** | **48** | **P001** | **-** |
| *^†^****NOTCH1*** | **chr9:139390649** | **NM_017617** | **fsdeletion** | **c.7541_7542delCT** | **p.P2514fs** | **13%** | **954** | **P111** | **-** |
| *^†^NOTCH1* | chr9:139390649 | NM_017617 | fsdeletion | c.7541_7542delCT | p.P2514fs | 54% | 2,479 | P114 | - |
| *^†^****NOTCH1*** | **chr9:139390649** | **NM_017617** | **fsdeletion** | **c.7541_7542delCT** | **p.P2514fs** | **46%** | **36** | **P016** | **-** |
| *^†^NOTCH1* | chr9:139390649 | NM_017617 | fsdeletion | c.7541_7542delCT | p.P2514fs | 60% | 1,879 | P062 | - |
| *^†^****NOTCH1*** | **chr9:139390649** | **NM_017617** | **fsdeletion** | **c.7541_7542delCT** | **p.P2514fs** | **12%** | **1,852** | **P071** | **-** |
| *^†^NOTCH1* | chr9:139390649 | NM_017617 | fsdeletion | c.7541_7542delCT | p.P2514fs | 58% | 3,499 | P080 | - |
| *^†^****PTPN6*** | **chr12:7067226** | **NM_080549** | **missense** | **c.1351G>A** | **p.V451M** | **51%** | **1,191** | **P016** | **rs62621988** |
| *^†^****SF3B1*** | **chr2:198267491** | **NM_012433** | **missense** | **c.1866G>C** | **p.E622D** | **14%** | **28,824** | **P132** | **-** |
| *^†^SF3B1* | chr2:198267491 | NM_012433 | missense | c.1866G>T | p.E622D | 49% | 11,601 | P056 | - |
| *^†^****SF3B1*** | **chr2:198267489** | **NM_012433** | **missense** | **c.1868A>G** | **p.Y623C** | **42%** | **11,120** | **P108** | **-** |
| *^†^SF3B1* | chr2:198267489 | NM_012433 | missense | c.1868A>G | p.Y623C | 10% | 13,019 | P086 | - |
| *^†^****SF3B1*** | **chr2:198267481** | **NM_012433** | **missense** | **c.1876A>T** | **p.N626Y** | **48%** | **19,047** | **P088** | **-** |
| *^†^****SF3B1*** | **chr2:198267480** | **NM_012433** | **missense** | **c.1877A>T** | **p.N626I** | **9%** | **3,760** | **P035** | **-** |
| *^†^****SF3B1*** | **chr2:198267480** | **NM_012433** | **missense** | **c.1877A>G** | **p.N626S** | **7%** | **8,908** | **P079** | **-** |
| *^†^****SF3B1*** | **chr2:198267371** | **NM_012433** | **missense** | **c.1986C>A** | **p.H662Q** | **45%** | **852** | **P015** | **-** |
| *^†^****SF3B1*** | **chr2:198267361** | **NM_012433** | **missense** | **c.1996A>G** | **p.K666E** | **6%** | **11,613** | **P101** | **-** |
| *^†^SF3B1* | chr2:198267361 | NM_012433 | missense | c.1996A>G | p.K666E | 51% | 3,169 | P122 | - |
| *^†^****SF3B1*** | **chr2:198267360** | **NM_012433** | **missense** | **c.1997A>T** | **p.K666M** | **48%** | **5,418** | **P057** | **-** |
| *^†^****SF3B1*** | **chr2:198266837** | **NM_012433** | **missense** | **c.2095C>G** | **p.Q699E** | **51%** | **3,254** | **P071** | **-** |
| *^†^****SF3B1*** | **chr2:198266834** | **NM_012433** | **missense** | **c.2098A>G** | **p.K700E** | **11%** | **360** | **P011** | **-** |
| *^†^SF3B1* | chr2:198266834 | NM_012433 | missense | c.2098A>G | p.K700E | 44% | 362 | P021 | - |
| *^†^SF3B1* | chr2:198266834 | NM_012433 | missense | c.2098A>G | p.K700E | 48% | 2,448 | P084 | - |
| *^†^****SF3B1*** | **chr2:198266834** | **NM_012433** | **missense** | **c.2098A>G** | **p.K700E** | **9%** | **4,504** | **P091** | **-** |
| *^†^****SF3B1*** | **chr2:198266822** | **NM_012433** | **missense** | **c.2110A>T** | **p.I704F** | **48%** | **5,892** | **P110** | **-** |
| *^†^****SF3B1*** | **chr2:198266821** | **NM_012433** | **missense** | **c.2111T>A** | **p.I704N** | **19%** | **1,651** | **P100** | **-** |
| *^†^****SF3B1*** | **chr2:198266821** | **NM_012433** | **missense** | **c.2111T>C** | **p.I704T** | **8%** | **719** | **P035** | **-** |
| *^†^SF3B1* | chr2:198266611 | NM_012433 | missense | c.2225G>A | p.G742D | 14% | 13,125 | P111 | - |
| *^†^****SF3B1*** | **chr2:198266611** | **NM_012433** | **missense** | **c.2225G>A** | **p.G742D** | **13%** | **2,079** | **P044** | **-** |
| *^†^SF3B1* | chr2:198266611 | NM_012433 | missense | c.2225G>A | p.G742D | 40% | 3,085 | P050 | - |
| *^†^****SF3B1*** | **chr2:198265476** | **NM_012433** | **missense** | **c.2681A>G** | **p.D894G** | **54%** | **631** | **P100** | **-** |
| ***^¥^****TP53* | chr17:7579719 | NM_000546 | fsdeletion | c.77delT | p.L26fs | 89% | 1,610 | P064 | - |
| ***^¥^****TP53* | chr17:7579718 | NM_000546 | fsdeletion | c.78delT | p.L26fs | 9% | 1,481 | P064 | - |
| ***^¥^****TP53* | chr17:7579719 | NM_000546 | missense | c.77T>C | p.L26P | 20% | 169 | P064 | - |
| *^§^TP53* | chr17:7579559 | NM_000546 | nonsense | c.128T>A | p.L43* | 6% | 417 | P110 | - |
| *^§^TP53* | chr17:7579361 | NM_000546 | missense | c.326T>G | p.F109C | 81% | 6,515 | P101 | - |
| *^†^****TP53*** | **chr17:7578526** | **NM_000546** | **missense** | **c.404G>A** | **p.C135Y** | **70%** | **18,906** | **P095** | **-** |
| ***^¥^TP53*** | **chr17:7578475** | **NM_000546** | **missense** | **c.455C>T** | **p.P152L** | **9%** | **17,762** | **P132** | **-** |
| *^†^****TP53*** | **chr17:7578406** | **NM_000546** | **missense** | **c.524G>A** | **p.R175H** | **16%** | **5,040** | **P095** | **rs28934578** |
| ***^¥^****TP53* | chr17:7578280 | NM_000546 | missense | c.569C>T | p.P190L | 10% | 11,695 | P067 | - |
| *^†^TP53* | chr17:7578265 | NM_000546 | missense | c.584T>C | p.I195T | 8% | 15,913 | P086 | - |
| *^§^TP53* | chr17:7578253 | NM_000546 | missense | c.596G>A | p.G199E | 58% | 386 | P032 | - |
| ***^¥^****TP53* | chr17:7578208 | NM_000546 | missense | c.641A>G | p.H214R | 11% | 3,170 | P111 | - |
| ***^¥^****TP53* | chr17:7578204 | NM_000546 | missense | c.645T>A | p.S215R | 97% | 2,456 | P085 | - |
| ***^¥^****TP53* | chr17:7578190 | NM_000546 | missense | c.659A>G | p.Y220C | 96% | 1,683 | P091 | - |
| *^§^TP53* | chr17:7577580 | NM_000546 | missense | c.701A>G | p.Y234C | 6% | 7,440 | P058 | - |
| ***^¥^TP53*** | **chr17:7577580** | **NM_000546** | **missense** | **c.701A>G** | **p.Y234C** | **5%** | **2,635** | **P067** | **-** |
| ***^¥^****TP53* | chr17:7577559 | NM_000546 | missense | c.722C>T | p.S241F | 97% | 4,136 | P119 | - |
| ***^¥^****TP53* | chr17:7577556 | NM_000546 | missense | c.725G>A | p.C242Y | 99% | 3,730 | P117 | - |
| *^§^TP53* | chr17:7577550 | NM_000546 | missense | c.731G>A | p.G244D | 7% | 3,609 | P112 | rs28934572 |
| ***^¥^TP53*** | **chr17:7577548** | **NM_000546** | **missense** | **c.733G>T** | **p.G245C** | **7%** | **21,069** | **P132** | **rs28934575** |
| *^†^TP53* | chr17:7577547 | NM_000546 | missense | c.734G>A | p.G245D | 10% | 6,840 | P040 | rs121912656 |
| ***^¥^****TP53* | chr17:7577538 | NM_000546 | missense | c.743G>A | p.R248Q | 98% | 4,972 | P082 | rs1144065 |
| *^†^TP53* | chr17:7577142 | NM_000546 | missense | c.796G>A | p.G266R | 13% | 9,074 | P115 | - |
| *^†^TP53* | chr17:7577142 | NM_000546 | nonsense | c.796G>T | p.G266* | 20% | 7,472 | P094 | - |
| *^†^TP53* | chr17:7577113 | NM_000546 | missense | c.824_825delinsTC | p.C275F | 6% | 5,282 | P040 | - |
| ***^¥^****TP53* | chr17:7577108 | NM_000546 | missense | c.830G>T | p.C277F | 13% | 18,021 | P105 | - |
| *^§^TP53* | chr17:7577105 | NM_000546 | missense | c.833C>G | p.P278R | 7% | 9,393 | P112 | - |
| ***^¥^****TP53* | chr17:7577036 | NM_000546 | fsinsertion | c.902_903insC | p.P301fs | 13% | 9,566 | P067 | - |
| ***^¥^****TP53* | chr17:7576897 | NM_000546 | nonsense | c.949C>T | p.Q317* | 13% | 3,408 | P132 | - |
| *^†^****XPO1*** | **chr2:61719471** | **NM_003400** | **missense** | **c.1711_1712delinsAT** | **p.E571I** | **24%** | **3,789** | **P052** | **-** |
| *^†^****XPO1*** | **chr2:61719472** | **NM_003400** | **missense** | **c.1711G>A** | **p.E571K** | **26%** | **570** | **P001** | **-** |
| *^†^XPO1* | chr2:61719472 | NM_003400 | missense | c.1711G>A | p.E571K | 51% | 4,247 | P130 | - |
| *^†^XPO1* | chr2:61719472 | NM_003400 | missense | c.1711G>A | p.E571K | 48% | 4,581 | P028 | - |
| *^†^XPO1* | chr2:61719472 | NM_003400 | missense | c.1711G>A | p.E571K | 44% | 1,955 | P037 | - |
| *^†^****XPO1*** | **chr2:61719472** | **NM_003400** | **missense** | **c.1711G>A** | **p.E571K** | **49%** | **9,922** | **P057** | **-** |
| *^†^XPO1* | chr2:61719472 | NM_003400 | missense | c.1711G>A | p.E571K | 45% | 5,696 | P068 | - |
| *^†^XPO1* | chr2:61719472 | NM_003400 | missense | c.1711G>A | p.E571K | 18% | 5,262 | P072 | - |
| *^†^XPO1* | chr2:61719472 | NM_003400 | missense | c.1711G>A | p.E571K | 52% | 3,721 | P080 | - |
| *^†^XPO1* | chr2:61719472 | NM_003400 | missense | c.1711G>A | p.E571K | 50% | 4,457 | P091 | - |
| *^†^XPO1* | chr2:61719472 | NM_003400 | missense | c.1711G>A | p.E571K | 49% | 4,781 | P094 | - |
| *^†^XPO1* | chr2:61719472 | NM_003400 | missense | c.1711G>A | p.E571K | 52% | 2,197 | P096 | - |
| *^†^****XPO1*** | **chr2:61719472** | **NM_003400** | **missense** | **c.1711G>C** | **p.E571Q** | **13%** | **3,121** | **P048** | **-** |
| ***^¥^***Deletion on the second allele; ^†^no FISH data available; *^§^*no chromosome deletions | | | | | | | | | |
